# Transcriptional Basis for Haustorium Formation and Host Establishment in Hemiparasitic *Psittacanthus schiedeanus* Mistletoes

**DOI:** 10.3389/fgene.2022.929490

**Published:** 2022-06-13

**Authors:** Enrique Ibarra-Laclette, Carlos Ariel Venancio-Rodríguez, Antonio Acini Vásquez-Aguilar, Alexandro G. Alonso-Sánchez, Claudia-Anahí Pérez-Torres, Emanuel Villafán, Santiago Ramírez-Barahona, Sonia Galicia, Victoria Sosa, Eria A. Rebollar, Carlos Lara, Antonio González-Rodríguez, Francisco Díaz-Fleisher, Juan Francisco Ornelas

**Affiliations:** ^1^ Instituto de Ecología A.C. (INECOL), Red de Estudios Moleculares Avanzados (REMAv), Xalapa, Mexico; ^2^ Instituto de Ecología A.C. (INECOL), Red de Biología Evolutiva, Xalapa, Mexico; ^3^ Investigador por Mexico-CONACyT en el Instituto de Ecología A.C. (INECOL), Xalapa, Mexico; ^4^ Departamento de Botánica, Instituto de Biología, Universidad Nacional Autónoma de Mexico (UNAM), Ciudad de Mexico, Mexico; ^5^ Centro de Ciencias Genómicas, Universidad Nacional Autónoma de Mexico, Cuernavaca, Mexico; ^6^ Centro de Investigación en Ciencias Biológicas, Universidad Autónoma de Tlaxcala, Tlaxcala, Mexico; ^7^ Laboratorio de Genética de la Conservación, Instituto de Investigaciones en Ecosistemas y Sustentabilidad (IIES), UNAM, Morelia, Mexico; ^8^ INBIOTECA, Universidad Veracruzana, Xalapa, Mexico

**Keywords:** transcriptome, parasitic plant, mistletoe, *Psittacanthus schiedeanus*, haustorium

## Abstract

The mistletoe *Psittacanthus schiedeanus*, a keystone species in interaction networks between plants, pollinators, and seed dispersers, infects a wide range of native and non-native tree species of commercial interest. Here, using RNA-seq methodology we assembled the whole circularized quadripartite structure of *P. schiedeanus* chloroplast genome and described changes in the gene expression of the nuclear genomes across time of experimentally inoculated seeds. Of the 140,467 assembled and annotated uniGenes, 2,000 were identified as differentially expressed (DEGs) and were classified in six distinct clusters according to their expression profiles. DEGs were also classified in enriched functional categories related to synthesis, signaling, homoeostasis, and response to auxin and jasmonic acid. Since many orthologs are involved in lateral or adventitious root formation in other plant species, we propose that in *P. schiedeanus* (and perhaps in other rootless mistletoe species), these genes participate in haustorium formation by complex regulatory networks here described. Lastly, and according to the structural similarities of *P. schiedeanus* enzymes with those that are involved in host cell wall degradation in fungi, we suggest that a similar enzymatic arsenal is secreted extracellularly and used by mistletoes species to easily parasitize and break through tissues of the host.

## Introduction

Parasitic plants latch onto other plants and feed off them, either indirectly from another plant *via* mycorrhizal fungi (mycoheterotrophs) or directly *via* modified roots called haustoria (the so-called haustorial parasites; Twyford, 2018; Nickrent, 2020). Approximately 1.8% of the more than 300,000 known flowering plant species are parasitic, with haustorial parasites (c. 4,800 species) having evolved at least 12 times independently across the angiosperms and showing extremely diverse morphologies, ranging from large trees to tiny herbaceous plants (Barkman et al., 2007; Westwood et al., 2010; Twyford, 2018; Nickrent, 2020). Depending on the site of attachment to the host, parasitic plants are classified into stem (aerial) or root parasites and based on whether these have retained or lost photosynthetic activities as hemiparasites or holoparasites, respectively (Nickrent and Musselman, 2004; Têšitel, 2016; Yoshida et al., 2016; Teixeira-Costa and Davis, 2021). Hemiparasites are photosynthetically active but derive water, minerals, nutrients, and significant amounts of carbon from their hosts, whereas holoparasites lack photosynthetic activity and rely entirely on a host for carbon (Poulin et al., 2011; Twyford, 2018; Nickrent, 2020; definitions of hemiparasites and other functional classifications reviewed in Têšitel, 2016; Teixeira-Costa and Davis, 2021).

Among the several parasitic angiosperm lineages, the sandalwood order, Santalales, is the only one that contains more than one family, having the largest number of species (2,428) among parasitic lineages, whereas Orobanchaceae (Lamiales) is the largest single parasitic flowering plant family (Nickrent, 2020). Each of these two lineages encompasses the widest array of nutritional modes among parasitic lineages, including autotrophic non-parasites, hemiparasites, and holoparasites (Nickrent, 2020). The aerial parasites of the Santalales, known as mistletoes, are not a monophyletic group, and thus the term “mistletoe” refers to a functional group that refers to all aerial or stem hemiparasitic species within the order (Watson, 2001; Nickrent, 2020). Mistletoes latch onto their host plant *via* a haustorium, which penetrates the host’s tissues, creates a vascular connection, and facilitates the transfer of water and nutrients, thereby forming a living physiological bridge between the host and the haustorial parasite (Cocoletzi et al., 2016, 2020; Teixeira-Costa, 2021a). However, the molecular understanding of plant parasitism is relatively in its infancy. Genome reduction due to loss of mitochondrial genes (Skippington et al., 2015; Li et al., 2017) that encode respiratory complex I (a main component of the energy production pathway in aerobic organisms), and other signs of degenerative evolution such as genome miniaturization and accelerated mutation rates have been documented in the Santalales (Xi et al., 2013; Molina et al., 2014; Petersen et al., 2015a,b; Fan et al., 2016). In parasitic plants whose complete plastomes have been studied, the selection patterns in plastid genes differ from those observed in other eudicots, with a relaxation of selection constraints across genes involved in photosynthesis (Wicke et al., 2013; Petersen et al., 2015b; Maclean et al., 2018). This apparent reverse evolution may be related to the fact that mistletoes (obligate parasites) require a host to derive nutrients for their survival and to complete their life cycle (Heide-Jørgensen, 2008). However, most of the stem hemiparasitic species in the Loranthaceae family produce chlorophyll and likely synthesize some of the nutrients by photosynthesis (Nickrent et al., 2010; Têšitel, 2016; Teixeira-Costa and Davis, 2021).

Genomic research is needed across a wide range of parasitic flowering plants to gain a better understanding of the evolution and function of parasitism (Petersen et al., 2015a; Li et al., 2017; Shin and Lee, 2018a,b). These studies can facilitate and accelerate our progress towards the molecular understanding of plant parasitism and its evolutionary consequences (Wang et al., 2016a; Wang et al., 2016b; Li et al., 2017; Su et al., 2021). Genomic resources in hemiparasitic plants can identify genes involved in developmental, morphological, and phenological changes, as well as candidate genes associated with the formation and specialization of haustoria. While great advances have been made into understanding parasitism genes, there are fundamental open questions as to the actual genetic changes necessary for parasitism, how (and when) these genes are expressed, and the way these genes interact to initiate attachment and form haustoria (Twyford, 2018).

Several recent studies have used next-generation RNA sequencing technology (RNA-seq) for many plant species to generate transcriptome information (e.g., Liu et al., 2020; Xu et al., 2020; Mei et al., 2021; Nguyen et al., 2021; Pérez-Torres et al., 2021). As the transcriptome actively changes depending on factors such as developmental stage and environmental conditions (e.g., Girke et al., 2000; Dong et al., 2004), researchers can determine when and where genes are turned on or off across types of cells and tissues. Thus, by studying the transcriptome of mistletoes, it would be possible to generate a comprehensive picture of which genes are active at various stages of host attachment and haustorial formation (e.g., Sánchez-Sevilla et al., 2017). However, mistletoes’ large genomes and changing seed germination phases have limited the utility of functional genomics and gene discovery approaches for gene identification. Some studies have shown the underlying mechanisms of haustoria development in parasitic plants. For example, a single electron reducing quinone oxidoreductase (TvPirin) is required to trigger the haustorium development in the roots of *Triphysaria versicolor* (Lamiales, Orobanchaceae; (Bandaranayake et al., 2010). Transcriptomics has been used to identify differentially expressed genes in the process of parasitism of *Cuscuta pentagona* (Solanales, Convolvulaceae), including genes encoding plant hormones (e.g., auxin, gibberellin, and strigolactones), transporters, and genes associated with cell wall modifications (Ranjan et al., 2014). Recently, small RNA sequencing has shown that microRNAs (miRNAs) in dodders’ (*Cuscuta* spp.) can target host genes (*Arabidopsis thaliana*) and improve parasitism (Shahid et al., 2018). The first haustoria transcriptome (Wei et al., 2020) of a Loranthaceae species, *Taxillus chinensis* (DC.) Danser, has shown that haustoria development in this hemiparasite likely involves genes encoding ribosomal proteins (RPs), transcription factors (TFs), ubiquitin, and disease-resistant proteins (DRPs). In turn, genes involved in cell wall metabolism, protein metabolism, mitochondrial electron transport, auxin signaling, and genes encoding nodulin-like proteins, appear to be important for haustoria development in the root parasite *Santalum album* (Santalales, Santalaceae) (Zhang et al., 2015). However, its seeds germinate and develop haustoria without the need for haustoria-inducing factors (Barrett and Fox, 1997; Nikam and Barmukh, 2009; Zhang et al., 2012). Thus, the molecular mechanisms of haustoria development remain mostly unknown for Loranthaceae stem parasites.

Here, we constructed the transcriptomic profile of haustoria development in the mistletoe *Psittacanthus schiedeanus* (Schltdl. and Cham.) G. Don (Loranthaceae) and report its complete chloroplast genome. We used transcriptomics to identify gene expression profiles of experimentally inoculated seeds across time. *Psittacanthus* is the most species-rich mistletoe genus of the family in the Americas (approximately 110 species; Kuijt, 2009; Dettke and Caires, 2021). *Psittacanthus schiedeanus* produces orange-to-yellow, self-compatible bisexual flowers pollinated mainly by hummingbirds (Supplementary Figure S1A; Ramírez and Ornelas, 2010), and ripe purplish-black, lipid-rich, one-seed fruits dispersed by a variety of birds (Supplementary Figures S1B–D; López de Buen and Ornelas, 1999; Ramírez and Ornelas, 2012). These hemiparasites are characteristic to canopy edges of cloud forests from northeastern Mexico to Guatemala (Ramírez-Barahona et al., 2017; Baena-Díaz et al., 2018), where they often parasitizes more than 20 host tree species, both native and non-native to cloud forests (López de Buen and Ornelas, 1999). In central Veracruz, Mexico, the most severe infections occur on deciduous (*Liquidambar styraciflua*, *Platanus mexicana*, *Acacia pennatula*) and evergreen (*Quercus germana*) host trees (López de Buen and Ornelas, 2002). Adult plants of *P. schiedeanus* are able to uptake water and xylem nutrients from both deciduous and evergreen host trees, suggesting they have the ability to modify their physiology according to the availability of host resources (Cocoletzi et al., 2016, 2020). Considering that differential expression likely underlies the processes of attachment and haustorial formation in these mistletoes, our results provide a comprehensive picture of how key genes are turned on or off, from seed inoculation to haustoria formation. Finally, we searched for glycoside hydrolases, molecules that are considered to be an important part of the cell wall-degrading enzymatic arsenal that mistletoes likely use to penetrate and parasite their hosts.

## Materials and Methods

### Plant Material and Growth Conditions

**FIGURE 1:**
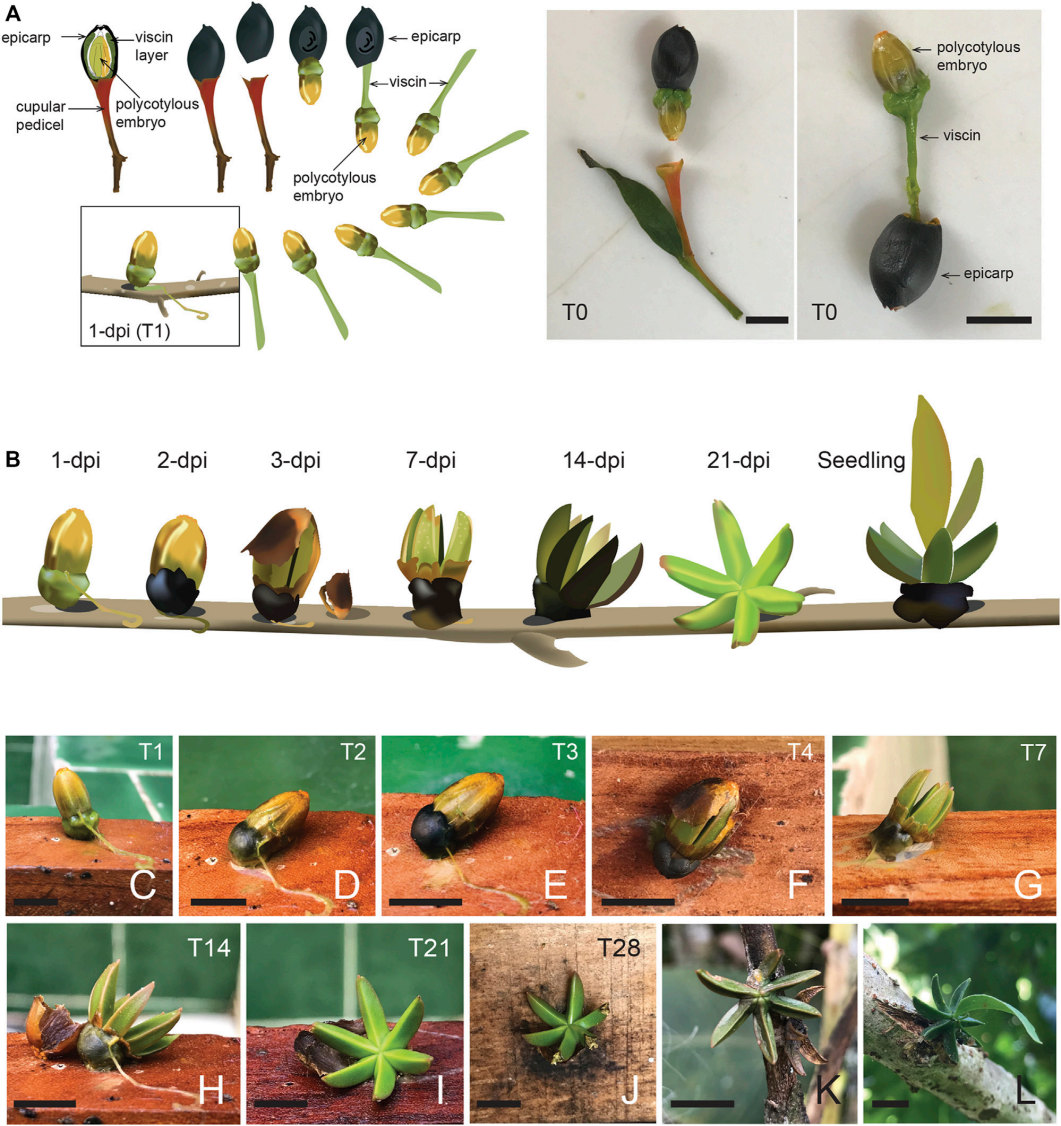
The *Psittacanthus schiedeanus* mistletoe seed inoculation experiment. **(A)** One-seeded ripe fruits had their epicarp manually removed, and seeds collected were then placed and glued with their viscin on wooden rectangle sticks (30–50 cm long) and placed at 5 cm from each other. Images show emerging embryo when manually pushed out before being placed on the wooden rectangle sticks aided by the green viscin and an extracted embryo with viscin at the base and epicarp removed. These are referred in the text as pre-inoculation, pre-sprouting seeds (time 0; T0). Morphology and anatomy of the infructescence and fruit are illustrated in Supplementary Figure S1. **(B)** Illustration of different development stages of inoculated seeds after 1–28 days after inoculation/germination (dai/dag) based on photographs. The plant material used for the RNA-seq study consisted of chlorophyllous material from the manually extracted seeds (T0), as illustrated in **(A)** and from four different development stages of the seeds formed by the polycotylous embryo (T1**–**T4: 7, 14, 21, and 28 dai/dag. **(C–F)** Images of inoculated seeds placed on the wooden rectangle sticks, from one to 4 days after inoculation/germination (T1**–**T4). Note how the viscin dries out, the base of the embryo becomes black (the scar-like dark-area that potentially includes the suspensor that is pushed aside by the emerging haustorial organ, which is not morphologically terminal, but it develops from the flanks of the apical dome; (Kuijt, 1970; Kuijt, 2009) and the embryo’s envelope or seed cover breaks about the middle after 4 days of seed inoculation. **(G**–**J)** Images of seeds after 7 (T7), 14 (T14), 21 (T21) and 28 (T28) dai/dag, with samplings taken for the RNA-seq study from T0, T1**–**T4, T7, T14, T21 and T28. After seven dai/dag the polycotylous embryo starts opening by day 7 (cotyledons spread) and becomes fully open by day 21. After 28 dai/dag, the prismatic lobes (6**–**7 cotyledons) of the star-shaped bodies (polycotylous embryo), and which maybe enclose the embryo, start to lose turgor and after a period, they perished. **(K)** Seed inoculated on a branch of a live host after 35**–**40 days. Note the putative leaf primordia at center of the star-shaped body. **(L)** Seedling with the first true leaves starting to emerge after 5 months of inoculation/germination. Photos by Juan Francisco Ornelas **(A**,**C–L)**. Scale bar = 0.5 cm. Illustrations by Julieta Ornelas Peresbarbosa **(A**,**B)**.

Seeds from *Psittacanthus schiedeanus* (mistletoe) plants growing on *Acacia pennatula* (Fabaceae) host trees were collected at La Pitaya, a cloud forest remnant with secondary riparian growth 6 km W of the city of Xalapa, Veracruz, Mexico (19°30′25″N, 96°57′39″W; at 1348 m above sea level). Seed production in the strict morphological sense does not occur in mistletoes because the reduction of ovary and ovules (Brown et al., 2010; Suaza-Gaviria et al., 2016). Functionally and ecologically, however, a mistletoe fruit is regarded here as a one-seeded fruit (Kuijt, 2009; Suaza-Gaviria et al., 2016). Ripe fruits were collected and fixed in FAA solution (Formaldehyde Acetic Acid Alcohol: 10% formaldehyde, 5% glacial acetic acid, 50% ethanol, and 35% distilled water), stored in 70% ethanol, and subsequently treated following the protocol of (Johansen, 1940). For light microscopy (LM), the fruit samples previously fixed and stored in 70% ethanol were dehydrated in a graded ethanol series (30, 50, 70, 85, 96 and 100%), cleared with xylene and embedded in Parawax^™^. For the observation of fruit anatomy, embedded specimens in wax were transversely and longitudinally sectioned with a rotary microtome and stained with safranin and fast green FCF or with toluidine blue and mounted (O’Brien and McCully, 1981), attached to coverslips and imaged and photographed. Morphological and anatomical aspects of the fruits were illustrated with the aid of photographs and microphotographs and camera lucida drawings. Digital images were processed and edited with the Adobe programs Photoshop CC and InDesign (further details in (Ornelas et al., 2022)). To perform seed germination experiments, additional ripe fruits had their epicarp manually removed, and seeds collected were then placed and glued with their viscin on 30 cm long wooden rectangle sticks (1.5 cm thick × 2.5 cm wide, approximately; Figure 1). Each seed was placed at 5 cm from each other. The plant material used for the RNA-seq study consisted of manually extracted seeds (time 0; T0), and four different development stages after seed inoculation/germination (T1–T4: 7, 14, 21, and 28 days after inoculation/germination (dai/dag; Figure 1). The different sampled parts/tissues were pooled for RNA extraction. That is, we collected the chlorophyllous star-shaped bodies whose prismatic lobes enclose the embryo lacking endosperm (polycotylous embryo; Supplementary Figure S1) according to descriptions (Kuijt, 1970, 2009; Kuijt and Hansen, 2015), and at the base of which it is possible to appreciate its corresponding developing haustorium (haustorial disk) (Figure 1). During the experiment (including the sampling days), the wooden sticks with manually glued seeds were maintained in a back porch/garden exposed to environmental conditions but daily watered with a spray bottle (moisture). The collected material was *in situ* frozen using liquid nitrogen, transported, and stored at −80°C until used. Once in the laboratory, the plant material was pulverized cryogenically using a mortar and pestle. Each biological replicate used in the present study (three in total) consisted of equivalent amounts (by weight) of the collected plant material once pulverized; in all cases, from at least three individual samples.

### RNA Isolation, Library Preparation, and Sequencing

For isolation of sufficient high-quality RNA from *P. schiedeanus*, 100 mg of pulverized tissue and the RNeasy kit (QIAGEN) were used following the manufacturer’s instructions. RNA concentration was measured on a NanoDrop 2000c spectrophotometer (Thermo Scientific) while RNA integrity was evaluated using both capillary electrophoresis by Bioanalyzer 2100 System (Agilent Technologies) and agarose electrophoresis. RIN (RNA Integrity Number) values varied from 8.5 to 9.5. Then, following the manufacturer’s instructions, high-quality RNAs samples were processed with the TruSeq RNA Sample Prep Kit version 2.0. Each library was independently labelled with a specific multiplexing index (Illumina) to identify each sample once performed the sequencing run. The RNA-seq libraries were generated and sequenced in the Massive Sequencing Unit of the Instituto de Ecología A.C. (INECOL, Veracruz, Mexico) using NextSeq500 platform (Illumina) and 2 × 150 bp paired-end reads format.

### Pre-Processing of Raw Data and Transcriptome Assembly

The fastq files obtained from the sequencing platform were subjected to a process in which once the sequence adapters (multiplexing indexes, Illumina) were trimmed, the low-quality paired-end reads were removed. Sequences with a minimum quality of 20 (Phred score) in 90% percent of the bases and an average quality value of 30 (Phred scores) calculated for the entire read, were selected as high-quality sequences and in consequence were processed by merging the paired reads with overlapping endings once the adapter sequences were removed. A Python-based script (qualityControl.py) and the SeqPrep version 1.1 program were used for these purposes, both freely available at Github (https://github.com/Czh3/NGSTools/blob/master/qualityControl.py and https://github.com/jstjohn/SeqPrep, respectively). Prior to the merging of paired reads and to improve the assembly quality and considerably reduce the computing timing, an *in-silico* normalization of paired-end reads was performed. A Perl-based script (insilico_read_normalization.pl), which is part of the tools package from the assembler (https://github.com/trinityrnaseq/trinityrnaseq/wiki/Trinity-Insilico-Normalization), was used for that purpose. The parameters used in this *in-silico* normalization process were a *k*-mer size of 25 bp and coverage of 50×. Merged and unmerged paired-end reads resulting were *de novo* assembled using the Trinity assembler (Grabherr et al., 2011). The resulting contigs (unique transcripts or uniGenes) were then analyzed with the SeqClean program (Chen et al., 2007). Poly A/T tails, ends rich in Ns (undetermined bases) and low complexity sequences were removed in this step.

### Cp-Like Sequences Screening, and Identification of Protein-Coding Regions Into Unique Transcripts Sequences (UniGenes)

Considering that the assembly process of chloroplast (cp) whole genome from RNA-seq data is a fast, accurate, and reliable strategy due to the almost full transcription of these chlorophyll-containing plastids, a screening of Cp-like sequences was performed before uniGenes annotation process (Osuna-Mascaró et al., 2018). BLASTClust program was used for this purpose considering as reference the *Elytranthe albida* chloroplast complete genome sequence (NCBI Reference Sequence: NC_045108.1). Cp-like sequences were considered if they showed an identity of at least 75% over 80% or more of the length of the sequence compared against the reference. In addition to Cp-like sequences, which were used to perform the assembly of *P. schiedeanus* chloroplast genome (see section below), the environmental-derived contaminant sequences were also removed from uniGenes set using DeconSeq software (Schmieder and Edwards, 2011). DeconSeq requires a pre-built database of potential contaminant sequences, and for this study it was generated considering angiosperm plants transposable elements (RepBase; http://www.girinst.org/repbase/) and the protein-coding transcripts present in the available and sequenced genomes of some insects, bacteria, and fungi (https://www.ncbi.nlm.nih.gov/genome/).

Remaining *P. schiedeanus* uniGenes were processed with AlignWise (Evans and Loose, 2015), a pipeline that drives several programs such as BLAST (Altschul et al., 1990), MUSCLE (Edgar, 2004), and GeneWise (Birney et al., 2004), to identify coding regions through a homology-based method that additionally corrects out-of-frame insertions/deletions. As reference in this process, almost 3 million genes (transcripts and their proteins) belonging to more than 80 angiosperm plant species whose genome is completely sequenced and available on GenBank database, were included. Only CDS which once translated generated peptides or proteins longer than 105 nucleotides (code for peptides or proteins of at least 35 amino acids) were kept and considered for the annotation process. Assembled transcriptome completeness was assessed using BUSCO software version 3.0.1 (Simao et al., 2015) and the predefined set of single copy (or with a reduced copy number) orthologous genes shared by species belonging to eudicotyledons clade (*n* = 1,375; https://busco.ezlab.org/busco_v4_data.html).

### Chloroplast Genome Assembly

The orientation and order of the Cp-like sequences obtained from *de novo* assembled transcriptome was performed by RaGOO pipeline (Alonge et al., 2019), a reference-guided scaffolder. *Elythranthe albida* chloroplast genome (Guo and Ruan, 2019; NC_045108.1) was used as reference. Additionally, and in order to compare the reliability of the assembly as well as to circularize and complete the chloroplast genome (fill the gaps), a still unpublished 100 bp-paired-end read dataset, was used. This dataset generated from genomic DNA which was provided by Dr. Juan Francisco Ornelas, was independently processed with NOVOPlasty assembler (Dierckxsens et al., 2017). A single circular high-quality whole chloroplast genome (a consensus sequence) was generated by the multiple sequences’ alignment in which both, the unique sequence generated by NOVOPlaty software as well as those Cp-like sequences obtained from the assembled transcriptome and processed with RaGOO were included. The annotation process was performed with CpGAVAS software (Liu et al., 2012).

### Homolog Genes Identification and Functional Annotation

*Psittacanthus schiedeanus* transcriptome annotation consisted of identification of homologs genes (similarity search) and the clustering of orthologs (and paralogs) genes shared between some representative angiosperm plant species (mostly belonging to the Pentapetalae clade). In addition, protein domains identification, and the functional classification based on GO terms assignment (Gene Ontology annotation) were also performed. The annotation process consisted of three nonexclusive sequential steps: *1*) homologs proteins search to the mistletoe genes followed by recognition of conserved protein domains, *2*) functional categorization based on gene ontology terms (GO-terms), and *3*) identification of orthologs genes across related plant species. First, the *P. schiedeanus* proteins (or peptides) resulting from translated uniGenes were compared with the complete set of proteins-coding genes of the following plant species: *Amborella trichopoda* Baill. (Amborellales, Amborellaceae); *Nymphaea colorata* Peter (Nymphaeales, Nymphaeaceae); *Papaver somniferum* L. (Ranunculales, Papaveraceae); [Asterids clade:] *Daucus carota* subsp. *sativus* (Hoffm.) Arcang. (Apiales, Apiaceae); *Helianthus annuus* L. (Asterales, Asteraceae); *Coffea arabica* L. (Gentianales, Rubiaceae); *Olea europaea* var. *sylvestris* L. (Lamiales, Oleaceae); *Solanum lycopersicum* L. (Solanales, Solanaceae); [Rosids clade:] *Arabidopsis thaliana* (L.) Heynh. (Brassicales, Brassicaceae); *Tripterygium wilfordii* Hook.f. (Celastrales, Celastraceae); *Cucumis melo* L. (Cucurbitales, Cucurbitaceae); *Glycine max* (L.) Merr. (Fabales, Fabaceae); *Juglans regia* L. (Fagales, Juglandaceae); *Populus trichocarpa* Torr. and A.Gray (Malpighiales, Salicaceae); *Theobroma cacao* L. (Malvales, Malvaceae); *Eucalyptus grandis* W.Hill ex Maiden (Myrtales, Myrtaceae); *Prunus persica* (L.) Batsch (Rosales, Rosaceae); *Citrus sinensis* (L.) Osbeck (Sapindales, Rutaceae); *Vitis vinifera* L. (Vitales, Vitaceae); and *Santalum album* L. (Santalales, Santalaceae). With only one exception (*A. thaliana*; https://www.arabidopsis.org/), all protein sets were downloaded from the latest version available on GenBank database (https://www.ncbi.nlm.nih.gov/). BLASTp algorithm and the single-directional Best Hit (SBH) method were used for this purpose (*e*-value 10^−5^). Second, using the pFam database (Punta et al., 2012; Finn et al., 2016; Mistry et al., 2021), Hidden Morkov Model (HMM)-based searches (Söding, 2005) were performed to identify proteins domains in the translated sequences of the *P. schiedeanus* uniGenes (*e*-value 10^−3^). Third, orthologs (and paralogs) genes shared between the above plant species and *P. schiedeanus* were identified by an *in-silico* analysis using OrthMCL version 2.0.9 pipeline (Li et al., 2003). This pipeline performs a bidirectional BLAST to identify homolog genes, then, considering an inflation value (Enright et al., 2002) (1.5 on this study) and using the Markov CLuster (MCL) algorithm (Enright et al., 2002), groups the orthologs genes (and paralogs) inferred across multiple taxa. We considered as a threshold an *e*-value of 10^−10^ in the BLAST step in which only proteins with minimum length of 30 amino acids were compared. This stringent cut-off value in the BLAST step required by OrthoMCL was chosen to avoid false-positive results. Finally, *P. schiedeanus* uniGenes were classified according to Gene Ontology (GO) terms into at least one of the three major categories (biological process, molecular function, and cellular components). These GO terms were inherited to *P. schiedeanus* genes mainly based on their identified *A. thaliana* homologs (ftp://ftp.arabidopsis.org/home/tair/Ontologies/).

### Phylogenetic Analysis

Phylogenetic relationships were resolved using the complete coding sequences from a total of 17 ortholog genes shared among the 21 angiosperm plant species selected as references (details in the section above). These 17 ortholog groups (OrthoGroups or OrthoMCL-defined protein families) were selected once confirmed that each corresponded to some of the genes belonging to the predefined set of 1,375 single-copy (or with a reduced copy number) genes conserved in the eudicots and which are used by BUSCO software version 3.0.1 (Simao et al., 2015) to assess the completeness of analyzed genomes/transcriptomes (details in the corresponding section above). Using MUSCLE algorithm (Edgar, 2004) implemented in Seaview program version 4.6.1 (Gouy et al., 2010), the sequences contained on these 17 OrthoGroups were aligned once those paralogs identified in some of the species compared were filtered out. In this manner, only single copies of each gene and from each species were included. Amino acid sequences were used to guide the alignment of their corresponding coding sequences. After using Trimal (Capella-Gutiérrez et al., 2009) to remove all positions with missing data, the four markers were concatenated in a single sequence representative for each species, and the best model for molecular evolution was identified for each gene using the corrected Akaike Information Criterion with PartitionFinder2 (Lanfear et al., 2017). The phylogenetic trees and their clade credibility values were inferred using MrBayes (Huelsenbeck and Ronquist, 2001; Ronquist et al., 2012) through a Markov chain Monte Carlo (MCMC) analysis of 2 runs over 1 × 10^6^ generations. The resulting tree was visualized using FigTree version 1.4.4 (http://tree.bio.ed.ac.uk/software/figtree/).

### Expression Profile Analysis of uniGenes and Gene Ontology (GO) Enrichment Analysis

The high-quality reads from each sampling point (T0–T4) were independently mapped onto the reference transcriptome (the annotated *P. schiedeanus* uniGenes) using the Bowtie2 software (Langmead and Salzberg, 2012). Subsequently, using the RSEM package (Li and Dewey, 2011) an expression profiles matrix was created containing each of *P. schiedeanus* uniGenes (rows) and the expected counts (EC) values calculated for each sampling point (columns). The EC values are representing the expression levels and are calculated by the maximum likelihood estimation approach as well as posterior mean estimates with 95% credibility intervals. These EC values are used by RSEM to calculate transcripts per million (TPM) and fragments per kilo base per million mapped reads (FPKM) values. Both measures considered as normalized values, can be used to represent the uniGenes expression levels. Here, TPM values were chosen to show the expression profiles because FPKM values are inconsistent among samples (Wagner et al., 2012). In addition, to identify differential expressed uniGenes (DEG) across sampling times, EC values estimated by RSEM were also processed with DESeq2 package (Love et al., 2014), which normalize and compare the data using likelihood ratio tests after performing negative binomial fittings. Data of inoculated/germinated seeds from each sampling time (T1–T4; 7, 14, 21, and 28 dag) were compared as pairs against the mistletoe’s pre-sprouting seeds (T0). UniGenes with False Discovery Rate (FDR)-corrected *p*-values ≤0.001 and a fold change values greater than 2 or less than 0.5 (Log_2_FC = ± 1), were considered as Differentially Expressed uniGenes (DEG). Then, a t-distributed stochastic neighbor-embedding (t-SNE) plot (van der Maaten and Hinton, 2008) was generated to perform a non-linear dimensional reduction in which DEGs with similar expression profiles were clustered close to each other in a lowdimensional space. To avoid a naïve selection and choose an appropriate number of clusters (*k*) in this *k*-means clustering analysis, the elbow method was used (Shi et al., 2021). In the elbow method the appropriate *k* value is defined by a plot in which the Sum of Squares Error (SSE) value show a significant (and elbow-shaped) decrease regarding to *k* values which are incremental. Finally, using g:Profiler software (Raudvere et al., 2019); https://biit.cs.ut.ee/gprofiler/gost), a gene ontology (GO) term enrichment analysis was performed for DEG. g:SCS was the selected multiple testing correction method and a *p*-value ≤0.05 defined as the significant threshold.

### Real-Time PCR (Quantitative Reverse Transcription PCR; qRT-PCR)

To validate the RNA-seq data, the expression pattern of 10 randomly selected DEGs was analyzed by qRT-PCR. The primers of the nominated uniGenes (Supplementary Table S1) were designed using Primer3 version 0.4.0 (http://bioinfo.ut.ee/primer3-0.4.0/). Actin (AT5G09810|UN063070) was used as an internal control or housekeeping gene in qRT-PCR (Jian et al., 2008; Jyothi Lekshmi et al., 2020); the reactions were performed in a STRATAGEN MX3005P (Agilent Technologies) real-time thermal cycler using SYBR Green^®^ PCR Master Mix (Life Technologies). The relative expression level of the selected DEGs was calculated with the 2^–ΔΔCt^ method (Livak and Schmittgen, 2001). Each reaction was performed in triplicate.

### Protein Modelling

The structural models from both, endo-polygalacturonase and endo-1,4-beta-D-mannanase of *P. schiedeanus* (members of GH28 and GH5-7 families, respectively), were generated by the rigid body grouping method using the SWISS-MODEL workspace (Arnold et al., 2006; http://swissmodel.expasy.org/). 3D protein structures that were used as templates were those that resulted as top-ranked after homologs search in the Protein Data Bank (PDB). To assess the accuracy of each of the modelled enzymes, they were checked by various parameters that included Z, GMQE (Global Model Quality Estimation) and QMEAN (Qualitative Model Energy ANalysis) scores. Once proteins were modelled, using UCSF Chimera program (Pettersen et al., 2004; Meng et al., 2006), main candidates were superimposed one by one with a well-characterized endopolygalacturonase from the phytopathogenic fungus *Fusarium moniliforme* (PDB ID: 1HG8; Federici et al., 2001), and a beta-mannanase from *Aspergillus niger* BK01 (PDB ID: 3WH9; Huang et al., 2014). The surface electrostatic potential or nonpolar to polar surface ratios in the active site, the distance between catalytic residues, as well as the global Root Mean Square Deviation (RMSD) of the superimposed 3D structures, were calculated also using UCSF Chimera program.

## Results

### Fruit Anatomy and Seed Germination

Ripe fruits of *P. schiedeanus* were collected during the 2020 fruiting season (December) from plants on *Acacia pennatula* for anatomical description (Supplementary Figures S1E–G) and to perform seed germination experiments (Figures 1A–L). When fruits are cut transversely or longitudinally, the star-shaped bodies (polycotylous embryo) are already formed inside (Supplementary Figures S1F,G). Fruit sections indicate the following parts: epicarp, viscin layer, seed cover, and the polycotylous embryo with six to seven cotyledons (Supplementary Figures S1E–G; further details in (Ornelas et al., 2022). Note that the basal section of the star-shaped body whose prismatic lobes (cotyledons) enclose the embryo (polycotylous embryo according to (Kuijt, 1970), which later lead to haustorium formation, is oriented upwards to the persistent calyx at the top of the fruit (not towards the base of the fruit and cupular pedicel; Supplementary Figure S1G). To provide gene expression profiles of developing haustoria through time, we conducted a germination experiment in which *P. schiedeanus* seeds were manually inoculated using their own viscin onto wooden rectangle sticks. We documented the development of the characteristic star-shaped chlorophylous bodies or seedlings (polycotylous embryos) during a month (Figure 1). Once the embryo is released from the seed cover, the cotyledons expand and open as flowers do. After 35–40 days, the first true leaves start to emerge from the center of the star-shaped body, but only if they have been inoculated on a live host (Figure 1); no haustorium-like structures are formed if they were inoculated onto wooden rectangle sticks. As sampling points to RNA-seq analysis (see methods for details), we chose five different stages of development: manually extracted seeds (T0) and four different developmental stages (T1–T4; and T7, T14, and T28), which represent the development of the polycotylous embryo at 7, 14, 21, and 28 days after seed inoculation/germination (dai/dag; Figure 1). These sampling points were chosen because the polycotylous embryos were completely released from the seed cover and cotyledons started to open after 7 days of inoculation (Figure 1).

### Quality Check, Preprocessing, and Construction of the uniGenes Set

Of the total paired-end reads generated (157,317,453), a high percentage (71.24%) passed the quality filters and were considered as high-quality reads, the remaining (28.76%) were removed (Supplementary Table S2). As expected, the *in-silico* normalization considerably reduced the reads number to include the assembly process in which only 11,487,568 reads were added (1,871,453 paired-end reads and 9,616,115 considered like single-end reads because they were joined through their overlapping regions; Supplementary Table S2). As a result of the assembly process conducted with Trinity (Grabherr et al., 2011), we generated a dataset comprising a total of 140,464 uniGenes after removing Cp-like, redundant, and environmental-derived contaminant sequences (*see* methods for details). The coding regions were identified, and out-of-frame insertions/deletions were corrected into it using a homology-based method implemented in AlignWise (Evans and Loose, 2015). Resulting proteins and/or peptides (ranging from 35 to 5,446 a.a, with an average length of 233.17 a.a.; Supplementary Figure S2A) were annotated as described below. According to an estimation of the completeness of the assembled transcriptome (*see* **Methods** for details), this dataset represents approximately 86% of the total protein-coding genes of *P. schiedeanus* genome (Supplementary Figure S2B).

### Assembly of Chloroplast Genome

We assembled the *Psittacanthus schiedeanus* chloroplast whole genome, which shows a typical circularized quadripartite structure with 115,023 bp containing one large single-copy region (LSC, 85,436 bp), one small single-copy region (SSC, 14,707 bp), and two inverted repeat regions (IRs, 22,147 bp); it incorporates 105 genes, including 74 protein-coding genes, 27 transfer RNA genes, and four ribosomal RNA genes (Supplementary Figure S3). Interestingly, the genome coverage was approximately 95% when considering only those uniGenes identified as Cp-like sequences that align to the reference (Supplementary Figure S3).

### UniGenes Annotation

**FIGURE 2:**
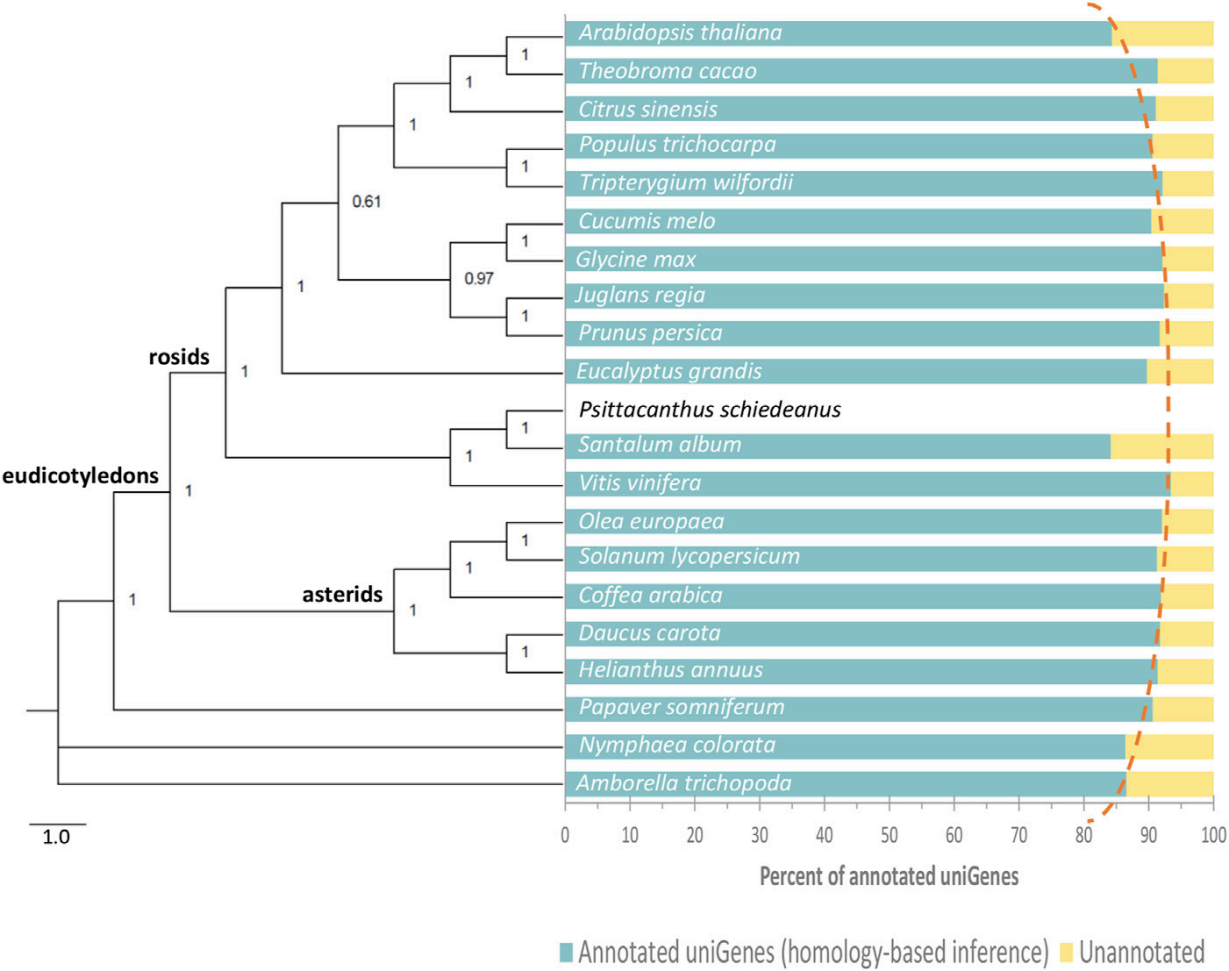
Phylogenetic tree and percent of *Psittacanthus schiedeanus* genes annotated by homology-based inference. The Bayesian phylogenetic tree (on the left) was constructed based on 17 single-copy orthologs nuclear genes (a total of 19,692 nt) shared among 21 angiosperm plant species analyzed. Numbers near the nodes indicate Bayesian posterior probabilities. On the right of the phylogenetic tree and for each plant species included, the percent of *P. schiedeanus* uniGenes that were annotated based on their homologous proteins. Homolog’s search was carried out by using the BLASTp algorithm (cut-off *e*-value of 10^−5^).

As expected, the number of *P. schiedeanus* uniGenes that were successfully annotated by homology with respect to the reference species (*see* methods for details) varied depending on the quality of the predicted gene models into the reference genomes and on their evolutionary relationships (Figure 2 and Supplementary Table S3); the number annotated increases as the reference species are phylogenetically more closely related (dotted red line, Figure 2). Regarding conserved protein domains, one domain and up to a maximum of seven were identified in a total of 88,611 uniGenes (63.08% from the total; Supplementary Table S4). The observed discrepancy between our data and that for the reference *S. album* species, which also belongs to the Santalales, is likely due to the poor quality and high fragmentation of *S*. *album*’s draft genome. In consequence, gene models predicted in *S. album* genome likely contain errors, including missing exons, non-coding sequence retention in exons, and fragmented or incomplete genes. Note that the evolutionary relationships among compared species in the present study were resolved based on the alignment of the complete coding sequences from 17 genes (Supplementary Table S5; 19,692 nucleotides) identified in all compared species corresponding to some of those single-copy genes which were also used to assess the completeness of the *P. schiedeanus* transcriptome (*see* **Methods** for details). Despite the strongly supported phylogenetic estimate, there are inconsistencies in the tree, mainly because *S. album* and *P. schiedeanus*, which belongs to Santalales order (superasterid), were grouped with *V. vinifera* (superrosid) (Cole et al., 2019; Zeng et al., 2017; Figure 2). Considering that even when some authors (Rokas et al., 2003) have claimed that the procedure of applying standard methods to concatenated multigene data leads to a strongly supported phylogenetic estimate, assumed to be the species tree; some authors have noted that differences in individual gene histories can cause the concatenation procedure to fail (Mossel and Vigoda, 2005). This, besides the low number of species included, could explain, at least in part, the strong support in the generated phylogenetic tree in the present study.

*Psittacanthus schiedeanus* uniGenes were also classified according to at least one of the following three major gene ontology (GO) slim categories: biological processes (BP), molecular functions (MF), and cellular components (CC). The subcategories assigned were obtained based mainly on the GO annotations available (ftp://ftp.arabidopsis.org/home/tair/Ontologies/) for *Arabidopsis thaliana* proteins, which were identified in the first step of the annotation process, as homologs of the mistletoe uniGenes. At least one GO term was assigned to a total of 62,749 mistletoe UniGenes, and the assignments included 827 unique GO terms from BP, 813 from MF and 375 from CC (Supplementary Table S6). Considering that more than one GO term can be assigned to a single gene, after functional categorization we estimated an average of two GO terms allocated to each of the mistletoe uniGenes (Supplementary Table S6).

In the last step of the annotation process, ortholog (and paralog) identification was conducted between *P. schiedeanus* and the other reference plant species used in this study (*see* **Methods** for full species names). A total of 1,009,348 proteins (29,711 from *A. trichopoda*, 22,571 from *A. thaliana*, 36,825 from *C. sinensis*, 64,563 from *C. arabica*, 27,864 from *C. melo*, 41,643 from *D. carota*, 41,237 from *E. grandis*, 70,131 from *G. max*, 70,323 from *H. annuus*, 43,625 from *J. regia*, 31,419 from *N. colorata*, 51,423 from *O. europaea*, 79,587 from *P. somniferum*, 49,534 from *P. trichocarpa*, 30,938 from *P. persica*, 105,202 from *P. schiedeanus*, 63,791 from *S. album*, 34,995 from *S. lycopersicum*, 29,374 from *T. cacao*, 45,778 from *T. wilfordii*, and 38,814 from *V. vinifera*) were grouped in a total of 68,069 OrthoGroups or OrthoMCL-defined protein families (Supplementary Table S7). Among these, 6,058 OrthoGroups were shared among all reference species (including *P. schiedeanus*); some of these OrthoGroups (7848, 10075, 10400, 10626, 10630, 11088, 11326, 11540, 11916, 12045, 12056, 12141, 12167, 12560, 12862, 12868, and 13351) were used to resolve the phylogenetic relationships among species (Figure 2 and Supplementary Tables S5, S7).

### Gene Expression Profile Changes During Development

To carry out the analysis of differential expression, we first performed a principal component analysis (PCA) to detect major sources of variance underlying the selected sampling points (T0–T4). Transcripts per million reads (TPM) values were chosen to show expression profiles (Supplementary Table S8). The two-dimensional PC plot with the first two principal components (PC1 and PC2) best illustrated the variance among expression profiles, with a proportion of explained variance of 22 and 11%, respectively (Supplementary Figure S4A). Since all libraries were independently included in the PCA, a hierarchical clustering tree of all libraries (Supplementary Figure S4B) indicates that the biological replicates have a high reproducibility and that the samples can be divided in at least two major groups: group 1 (T0) that represents pre-sprouting seeds, and group 2 that includes the remaining sampling points (T1–T4) representing the polycotylous embryos at 7, 14, 21, and 28 days after seed inoculation/germination. Based on these results, pair-wise comparisons (e.g., T1 vs T0, T2 vs T0, and so on) were performed to identify Differentially Expressed Genes (DEGs) involved in the development of polycotylous embryos. The RSEM software was used to estimate the expression levels from each uniGene on each sampling point and DEseq2 R package to calculate differential expression between them (*see* methods for details). In total, there were 2,096 uniGenes with two-fold or greater (Log_2_FC = ±1) differential expression and an adjusted significant *p*-value of ≤0.001 (Supplementary Table S9). Venn diagram comparison of DEGs shows that there is a similar percentage of up- and down-regulated uniGenes (53.9 and 65.3%, respectively; Supplementary Figure S4C), which modulate its expression during development of polycotylous embryos. Some of these DEGs appear to change their transcription level only at a specific time, while others, once up- or down-regulated, show only weak changes or trends through time (Supplementary Figure S4C). Interestingly, few DEGs are contra-regulated, i.e. genes showing opposite expression patterns when the FC contrast values are compared to each other (e.g., T1/T0 vs T2/T0; Supplementary Figure S4C).

**FIGURE 3:**
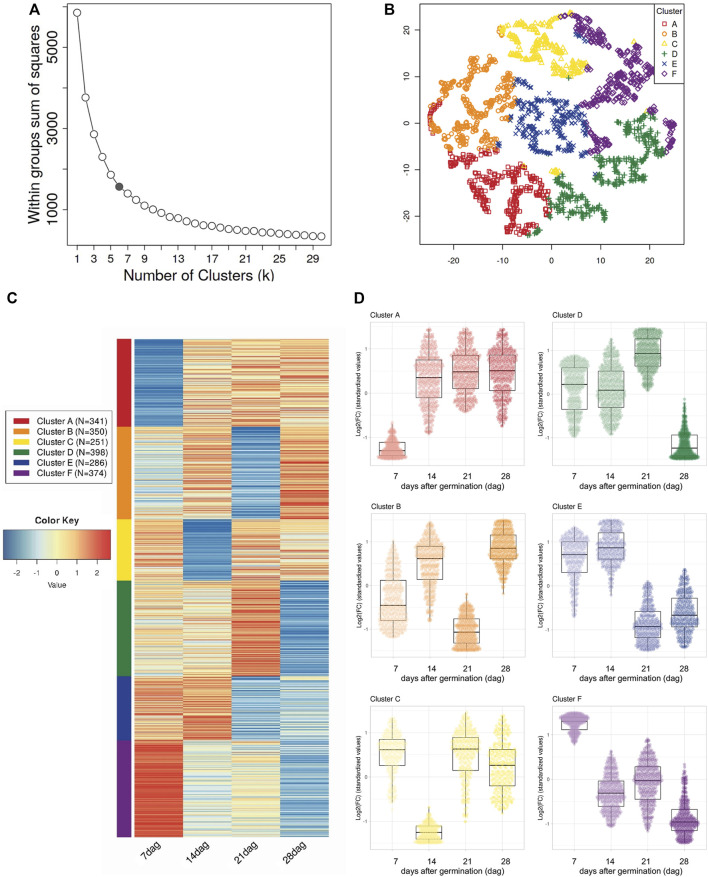
Expression patterns of Differentially Expressed uniGenes (DEG) identified in the transcriptome of *Psittacanthus schiedeanus* and involved in the compound endosperm development. **(A)** Elbow criterion applied over the curve of within-class sum-of-squares per number of clusters. The gray point is considered the elbow (*k* = 6). **(B)** t-SNE plot which shows clustering of 2,096 DEGs. The uniGenes coordinates are based on t-SNE dimensionality reduction according to the six expression profile categories (Clusters A–F) defined by the elbow method. uniGenes are represented by distinct marks shape and colored according to their cluster membership. In **(C)**, a heatmap (Log_2_-transformed Fold Change (FC) values) of DEG and belonging to each of the six distinctly defined clusters and represented in t-SNE map. As indicated at the bottommost, each column in the heatmap corresponds to each of the sampling points or days after germination (dag) [T1 (7 dag); T2 (14 dag); T3 (21 dag); T4 (28 dag)]. The cluster color code is on the left of the heatmap. The color scale bar indicates up (red) or down-regulation (blue) on each compound endosperm development stage (T1–T4) relative to the T0 (the mistletoe pre-sprouting seeds). Finally, in **(D)** panel, box plots depicting the expression pattern of DEG on each of the defined clusters (Clusters A–F; top to bottom, left to right). The median (middle quartile) marks the mid-point of the data and is shown by the line that divides each of the boxes into two parts. The top and bottom whiskers indicate the maximum and minimum expression values, respectively, excluding outliers. In this panel, dots (uniGenes) were colored according to each of the expression profile categories defined by t-SNE algorithm while their intensities (light to dark in color) represent the timeline of sampling points (7, 14, 21, and 28 dag, respectively).

According to these observations, the DEGs were grouped based on similarities in their expression patterns into six clusters using the Elbow method. These groups were visualized by the t-distributed stochastic neighbor embedding (t-SNE) reduction technique followed by running the *k*-means clustering algorithm and generated the corresponding heatmap (Figures 3A–C and Supplementary Table S9). From DEGs presumably involved in the development of the polycotylous embryo (Figure 3D), cluster A included 341 uniGenes with low levels of expression at seven dag but with a tendency to increase over time, clusters E and F (286 and 374, respectively) showed down-regulated tendencies but with slight differences, cluster E uniGenes (286) decreasing in their expression levels mainly at 21 and 28 dag and cluster F uniGenes (374) down regulated across analyzed times. In contrast, cluster D uniGenes (398) were mainly up regulated from 7 to 21 dag but levels of expression drastically decreasing at the end (28 dag). The expression patterns of the two remaining clusters were dynamic, with up- and down-regulation through time in cluster B (350 uniGenes) and a significant drop in expression only at 14 dag in cluster C (251 uniGenes).

### Real-Time PCR (Quantitative Reverse Transcription PCR; qRT-PCR)

We randomly selected 10 *P. schiedeanus* uniGenes to evaluate the validity of the RNA-seq data by qRT-PCR. The expression profiles obtained by qRT-PCR and RNA-seq data coincide in all sampling points analyzed (Supplementary Figure S5). This strongly suggests that RNA-seq data and subsequent interpretations are reliable.

### Functional Enrichment Analysis of Differentially Expressed uniGenes

**FIGURE 4:**
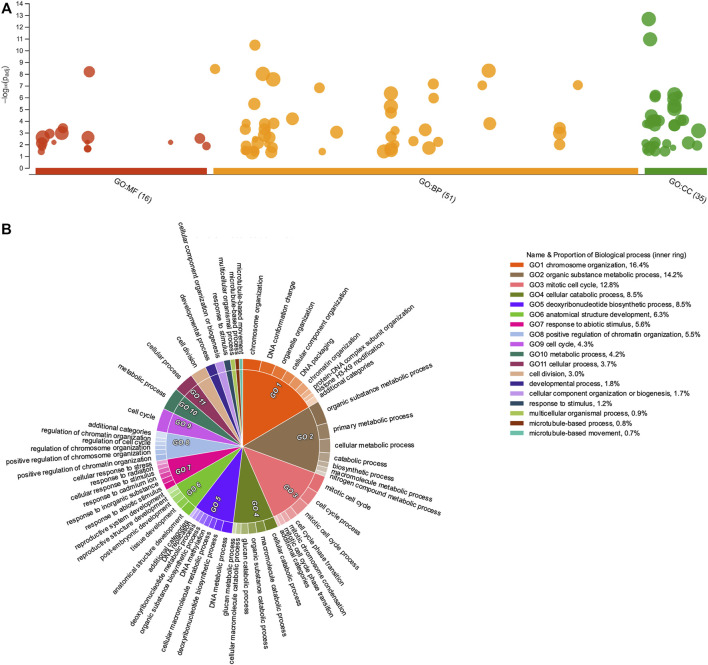
Gene Ontology enrichment of upregulated *Psittacanthus schiedeanus* uniGenes and involved in compound endosperm development after seed germination. **(A)** Manhattan plot illustrating the GO enrichment analysis results separated into the three major categories: MF (molecular functions), BP (biological processes), and CC (cellular components). The number in the source name in the *x*-axis labels shows how many GO terms were significantly enriched (g:SCS threshold, *p*-value ≤0.05). **(B)** Biological processes CirGO visualization plot. Intensity shades of colored categories reflect hierarchical relations between GO classes that were found enriched. Each subclass is represented in the plot by a white divisor line within each parent class, i.e., solid colors of the central pie charts. Features identities and names of all enriched functional categories (GO-terms) are reported in Supplementary Table S10.

**FIGURE 5:**
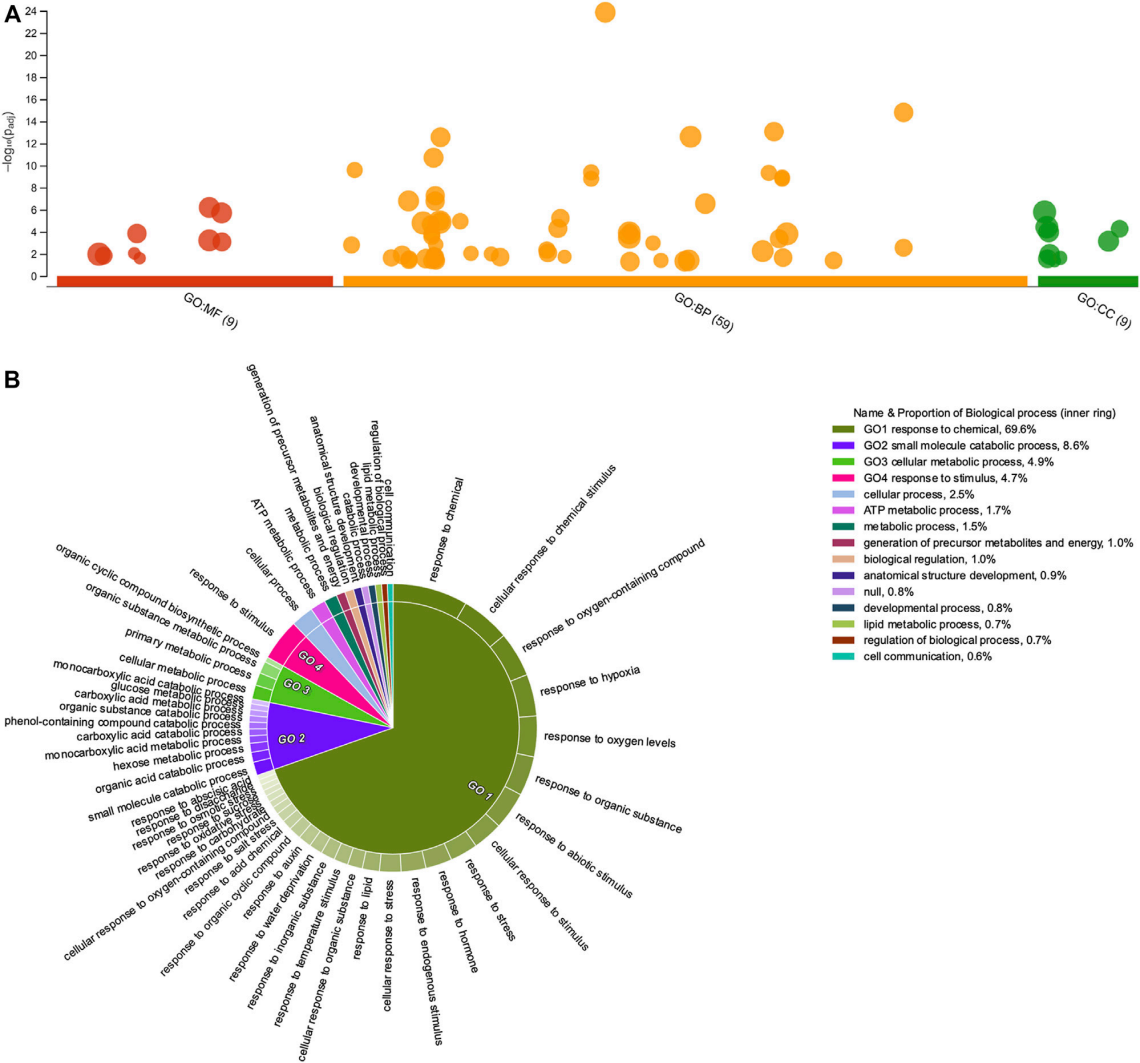
Gene Ontology enrichment of downregulated *Psittacanthus schiedeanus* uniGenes involved in compound endosperm development after seed germination. **(A)** Manhattan plot illustrating the GO enrichment analysis results separated into the three major categories: MF (molecular functions), BP (biological processes), and CC (cellular components). The number in the source name in the *x*-axis labels shows how many GO terms were significantly enriched (g:SCS threshold, *p*-value ≤0.05). **(B)** Biological processes CirGO visualization plot. Intensity shades of colored categories reflect hierarchical relations between GO classes that were found to be enriched. Each subclass is represented in the plot by a white divisor line within each parent class, i.e., solid colors of the central pie charts. Features identities and names of all enriched functional categories (GO-terms) are reported in Supplementary Table S10.

GO-terms enrichment analyses were done under two different, complementary assumptions: *1*) identification of enriched GO-terms considering the list of DEGs and classifying these into up- or down-regulated genes according to their FC values (≥2 or ≤0.5 (Log_2_FC = ±1)) at least in one time point (7, 14, 21, or 28 dag); *2*) identification of enriched categories by grouping uniGenes in each of the six clusters resulting from the *k*-means clustering analysis. Under the first assumption, GO-terms enriched in both up- and down-regulated DEG, revealed striking differences in the number of GO-terms associated with molecular functions [MF] and cellular components [CC]. However, GO-terms associated with biological processes [BP] were the most illustrative (Figures 4A, 5A, respectively and Supplementary Table S10).

Like other enrichment analyses, the information provided by GO-terms is structured as directed acyclic graphs with a clearly defined hierarchical structure, namely, a gene annotated with any GO-term is also annotated with every GO-term that is an ascendant, or parent GO-term, of the more specific GO-term; thus, each GO category will contain all the genes from each of its progeny’s categories. In consequence, major categories which often group most genes are more generalist and as such, less informative or even redundant at times. To simplify and visualize the enriched GO-terms in a two-dimensional hierarchically structured level, the lists of enriched GO terms with their g:SCS-adjusted *p*-values were inputted into REVIGO (Supek et al., 2011). Then, CirGO (Circular Gene Ontology) software (Kuznetsova et al., 2019) was used to visualize biological processes for up- and down-regulated uniGenes (Figures 4B, 5B, respectively and Supplementary Table S10). Interestingly, for the up-regulated uniGenes, the enriched GO-terms comprise biological processes such as “organelle organization” (GO:0006996); “chromosome organization” (GO:0051276) and “DNA packaging” (GO:0006323); other processes such as “cell division” (GO:0051301), “cell cycle” (GO:0007049) and its phase transitions (GO:0044772), are also well represented.

Regarding down-regulated uniGenes, the enriched GO-terms comprise two other biological processes: the “small molecule catabolic process” (GO:0044282) and the “cellular responses to chemical stimulus” (GO:0070887). The latter comprises subcategories such as “response to hormones” (GO:0009725), which include both “response to auxin” (GO:0009733) and “response to abscisic acid,” “response to temperature stimulus” (GO:0009266), “response to water deprivation” (GO:0009414), and “response to oxidative stress” (GO:0006979). When GO enrichment analyses were performed for each of the six clusters identified based on their expression patterns (Supplementary Figure S6), the “response to hormone” category only appears significantly enriched in the DEGs lists belonging to clusters A and D, uniGenes show initial low levels of expression (at seven dag) in the expression profile but with a tendency to increase through time (at 14 and 21 dag). The difference between these two uniGenes clusters in the expression profiles lies at 28 dag when uniGenes belonging to Cluster D show a decrease in their transcript level after a sustained increase.

### Auxin and Jasmonic Acid: Biosynthesis and Signaling by Proteins Involved

By carefully analyzing the lists of DEGs, we noticed that a considerable number of these uniGenes were annotated in functional categories related to auxin and jasmonic acid (JA). These GO categories, in addition to biosynthesis and signaling, consist of homeostasis and responses activated by these phytohormones (Supplementary Tables S11, S12). It is worth mentioning that a high percentage (>50%) of these DEGs were grouped into expression clusters A and D (Figure 3D). Interestingly, on the list of *P. schiedeanus* DEGs, we identified orthologs/homologs of Amidase 1 (AMI1; AT1G08980) and SUPERROOT 2 (SUR2; AT4G31500) (UN018669, UN042299, respectively). With one exception (TAA1; AT1G70560), we also found orthologs to all remaining functionally characterized enzymes involved in the auxin biosynthesis pathways (Supplementary Figure S7). The increasing tendency in AMI1 transcript levels during days 7–21 (cluster D), suggests that the Indole-3-acetamide pathway (AMI) (but not the Indole-3-pyruvic acid (IPyA) or Tryptamine (TAM) pathways) could be, to a large degree, the main contributor to the requirements of this phytohormone during early stages of development. In relation to JA biosynthesis pathway, in the *P. schiedeanus* uniGene collection, orthologs to all enzymes involved were found and some of them were also identified as DEGs (Supplementary Figure S7 and Supplementary Table S12).

Auxin and JA signaling pathways are similar (Supplementary Figure S7). Proteins such as those required for the ubiquitination process (E1–E3; Kliza and Husnjak, 2020) and CUL1 (orthologs to UN045078, UN114279), which is part of the co-receptor complex that triggers subsequent degradation of the repressors involved (IAA/JAZ), are shared by both signaling pathways (Supplementary Figure S7). Most of the *P. schiedeanus* uniGenes involved in auxin and JA signaling pathways were differentially expressed (Supplementary Figure S7 and Supplementary Tables S11, S12).

### Auxin and Its Role in Haustorium Formation

**FIGURE 6:**
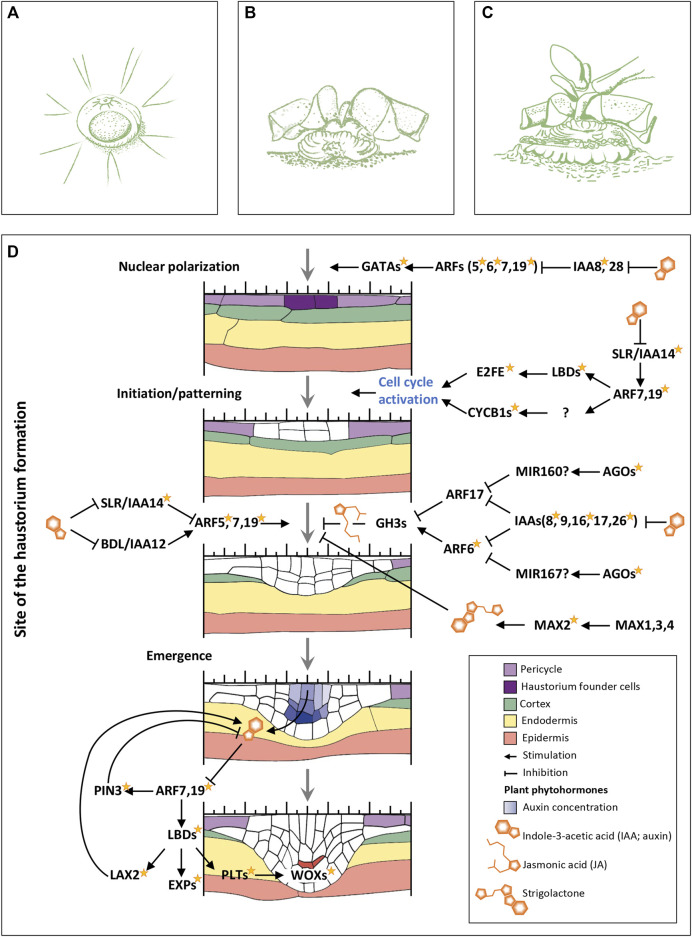
Germination in *Psittacanthus schiedeanus* and genes network presumably involved in haustorium formation. **(A)** Seedling inverted which show the emergence of the (oval) primary haustorium just below the small suspensor scar. **(B)** Older seedling showing the grooved haustorial cushion formed upon penetration. **(C)** Established seedling with early primary leaves. The tips of prismatic lobes of star-shaped bodies are removed in **(B**,**C)** (Re-drawn from Kuijt, 1970, 2009; Drawings by Julieta Ornelas Peresbarbosa). **(D)** Gene’s network regulating intrusive organ formation (haustorium) in *P. schiedeanus*. Different colors denote different tissues. Initiation and emergence rely on gene networks regulated by auxin (IAA), jasmonic acid (JA), and strigolactones. The yellow star next to the name of some proteins indicates that they were identified as DEGs. Genes/proteins with a question mark refer those that were not identified into the uniGenes collection generated in the present study. Abbreviations: GATAs, members of GATA family of transcription factors, zinc finger DNA binding proteins that control the development of diverse organs and tissues; ARFs, members of the auxin response factor family; IAAs, transcription regulators acting as repressors of auxin-inducible genes; LBDs, LOB domain-containing proteins; E2FE, E2F-like protein, an inhibitor of the endocycle, preserves the mitotic state of proliferating cells by suppressing transcription of genes that are required for cells to enter the DNA endoreduplication cycle; CYCB1s, Cycling family proteins; AGOs, ARGONAUTE family proteins; GH3s, Auxin-responsive GH3 family proteins; MAX2, MORE AXILLARY BRANCHES 2, members of the F-box leucine-rich repeat family of proteins; MAX1, MORE AXILLARY BRANCHES 1, members of the CYP711A cytochrome P450 family; MAX3,4, MORE AXILLARY BRANCHES 3,4 encodes proteins with similarity to carotenoid cleaving deoxygenases; PIN3, a regulator of auxin efflux, involved in differential growth; LAX2, a member of the AUX1 LAX family of auxin influx carriers; EXPs, expansins; PLTs, PLETHORA proteins; WOXs, WUSCHEL related homeobox proteins. Expression profiles of each of *P. schiedeana* uniGenes considered as orthologs (and paralogs) to each protein represented in the figure are shown in Supplementary Table S14.

*Psittacanthus schiedeanus* parasitizes through haustorium formation as most other parasitic plants do. In terms of its function, the haustorium resembles roots since it attaches the plant to a substrate (a host plant) for water and nutrient uptake. In *P. schiedeanus*, haustorium formation occurs during the analyzed seed germination stages (T1–T4; Figure 1), in the basal region of the star-shaped chlorophyllous bodies, while these emerge and develop from the seeds (Figures 6A–C and Supplementary Figure S8). Even though the physiological aspects of haustorium development in *Psittacanthus* species were first described in the 1970s, the molecular mechanisms that regulate its formation are still unknown. Based on these observations, we analyzed once again the list of DEGs but this time by surveying whether known molecular mechanisms that regulate the adventitious (ARs) or lateral roots (LRs) formation processes, could also be involved, at least in part, in the haustorium formation.

We performed an extensive search of genes involved in roots development and LRs and ARs formation using previous references (Bellini et al., 2014; Banda et al., 2019; Santos Teixeira and Ten Tusscher, 2019; Li, 2021). All genes, one-by-one, mentioned on these references (either *A. thaliana* or other plant species also included in our analysis) were identified into the OrthoMCL-defined protein families. The corresponding *P. schiedeanus* uniGenes grouped as orthologs to these reference genes were considered as candidates involved in haustorium formation especially if they were also identified as DEGs (Supplementary Tables S9, S13). Based on yielded number of genes, we identified a complex gene regulatory network that might be involved in haustorium formation, highly similar to the early steps of ARs and LRs formation (Figure 6D and Supplementary Table S13). Like ARs and LRs formation, the first intrusive organs formed, which give rise to the haustorial organ, require, and depend on gene networks regulated mainly by auxin, but also by jasmonic acid (JA) and strigolactones (Figure 6D). *Psittacanthus schiedeanus* orthologs were found in the uniGenes collection, and most of them were also identified as DEGs (Figure 6D and Supplementary Tables S3, S7, S9, S11–S13). Despite it has been discussed that most miRNAs are species-specific and of low abundance, it is also true that some of them are highly conserved across species (Chávez Montes et al., 2014). We suggest that in the haustorium formation model, which is very similar to that described for lateral roots formation, miRNAs participation is fundamental and should be further investigated.

### Glycoside Hydrolases Presumably Involved in Cell Wall Polysaccharide Degradation

We searched for plant cell wall degrading enzymes in the *P. schiedeanus* transcriptome. First, we identified all homologs/orthologs uniGenes to *A. thaliana* glycoside hydrolases enzymes (Minic and Jouanin, 2006). For downstream analysis, only those sequences in which the characteristic domains were also recognized were considered by searching into the pfam database (*e*-value ≤10^−3^). All these *P. schiedeanus* uniGenes were classified according to CAZy database (http://www.cazy.org/) which organizes the glycoside hydrolase (GH) families based on structurally related catalytic and carbohydrate-binding modules (or functional domains) present on enzymes that degrade, modify, or create glycosidic bonds (Drula et al., 2021). In addition, subcellular localization from each of these *P. schiedeanus* enzymes was predicted using Deep Loc v1.0 program (Almagro Armenteros et al., 2017). In total, 677 *P. schiedeanus* uniGenes were classified into 12 glycoside hydrolase (GH) families (and five subfamilies) and, interestingly, several members of these families (62.48%) seem to be soluble enzymes that are extracellularly secreted (Supplementary Table S15). Despite the high number of GH enzymes identified in the *P. schiedeanus* transcriptome, here we only analyzed in detail the soluble and extracellularly secreted enzymes from 28-family (GH28) and 5-family (7-subfamily; GH5-7). In fungi, the participation of these enzymes in the parasitic process has been proved and, additionally, some 3D structures are available in the PDB database and the specific motifs (and amino acid residues) comprising the catalytic site are known (Kester and Visser, 1990; Sakon et al., 1996; Hilge et al., 1998; van Santen et al., 1999; Armand et al., 2000; Federici et al., 2001; Dias et al., 2004; Huang et al., 2014; Zhou et al., 2014).

**FIGURE 7:**
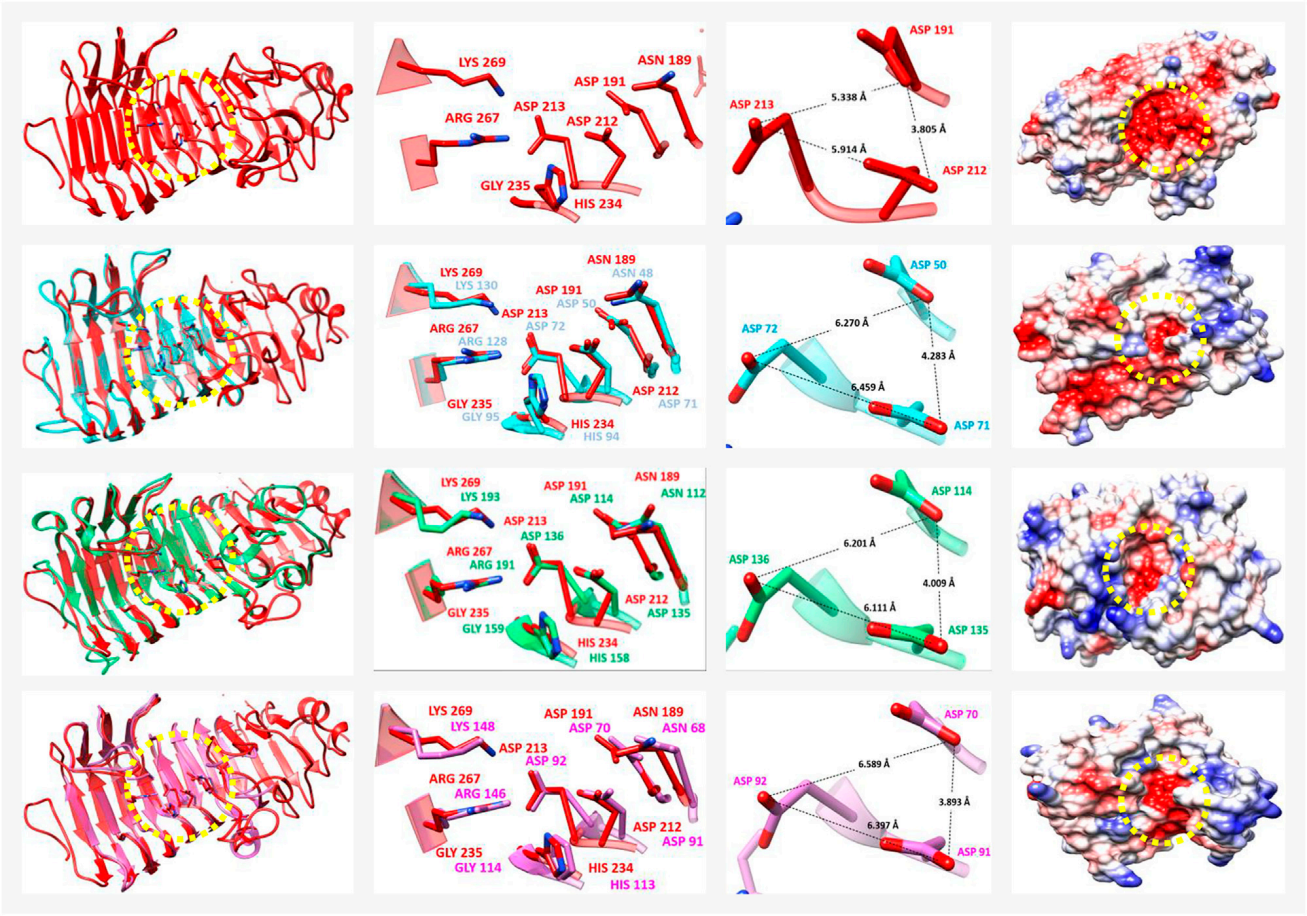
3D structure from some polygalacturonases (PG) members of GH28 family. From top to bottom, the PG from the phytopatogenic fungus *Fusarium moniliforme* (reference protein, PDB ID: 1HG8; Federici et al., 2001)) followed by the modelled PG of the mistletoe *Psittacanthus schiedeanus*. The PG from *P. schiedeanus* [UN045719, UN068437, and UN097465; cyan, green, and purple, respectively] were superimposed one by one with reference protein (red). From left to right (besides the 3D structures), a magnified image with the highly conserved amino acid residues which form the cleft of the active site, is shown. This image is followed by others with the estimated distance between the catalytic residues (three aspartic acid (Asp) residues) and finally, an electrostatic surface charge representation. The yellow dotted circles show the active site with a relatively low presence of positive charges. After alignment and the superimposing of the 3D modelled structures, the equivalent position of every amino acid residue into the active site is shown. PG 3D proteins models from *P. schiedeanus* were constructed *in silico* based on the homology to cited proteins and their solved crystal structure.

GH family 28 includes enzymes with endo-polygalacturonase (endo-PG), exo-polygalacturonase (exo-PG), or rhamnogalacturonase (RGase) activity; enzymes catalyze the hydrolysis of α-1,4-linked galacturonic acid units with an inversion of the configuration of the anomeric carbon atom. There are at least four motifs that form the catalytic site in these enzymes (motif I: SPNTDG; II: GDDC; III: CGPGHGISIGSLG; and IV: RIK) and they are conserved in plants, bacteria, and fungi (Kester and Visser, 1990). Some specific amino acid residues into each of these motifs are highly conserved and it has been proved by site-directed mutagenesis that they are essential for the activity of the plant cell wall degrading enzymes of phytopathogenic fungi (Kester and Visser, 1990; van Santen et al., 1999; Armand et al., 2000). Therefore, we compared 134 soluble and extracellularly secreted enzymes of *P. schiedeanus* (members of the GH28 family), with fungal enzymes, including those of which three-dimensional (3D) structures were available [*Aspergillus parasiticus* (Uniprot: P49575), *A. oryzae* (Uniprot: P35335), *A. niger* (PDB ID: 1CZF), *A. aculeatus* (PDB ID: 1IA5), and *Fusarium moniliforme* (PDB ID: 1HG8)]. Motifs I–IV were present in most of the analyzed *P. schiedeanus* uniGenes. However, amino acids that are highly conserved residues in enzymes from phytopathogenic fungi are conserved only in 13 of the *P. schiedeanus* uniGenes (Supplementary Figure S9). The aligned sequences revealed that homology percentage between mistletoe and fungi proteins ranged from 11.2 to 48.5% (Supplementary Table S16). The highly conserved amino acid residues present in the motifs from both, *P. schiedeanus* uniGenes and phytopathogenic fungi enzymes, are not only the residues involved in the hydrolysis of the α-1,4-glycosidic linkages, but are also some of those that make possible the formation of the active site cleft, in which the presence of positive charges if relatively low (van Santen et al., 1999; Federici et al., 2001; Supplementary Figure S9). The topology of the active site (cleft form and no tunnel form) and the electrostatic potential (negative) was confirmed in 9 of 13 of the *P. schiedeanus* uniGenes above mentioned; the four remaining enzymes show a tunnel form in the active site (Supplementary Table S17). These features were evaluated from the 3D structure of each *P. schiedeanus* enzymes that were generated using the rigid body grouping method of the SWISS-MODEL workspace and choosing the homologs proteins with the best BLAST scores as suitable templates for modeling (*see* methods for more details). The 3D structural model superposition of the *P. schiedeanus* enzymes with the endopolygalacturonase from the phytopathogenic fungus *Fusarium moniliforme* (PDB ID: 1HG8; Federici et al., 2001) show that the global Root Mean Square Deviation (RMSD) of these enzymes is in the range of 0.833–1.838 Å (Supplementary Table S17), which indicates that the predicted 3D structures are similar to the reference enzyme. Together, all these results strongly suggest that at least nine *P. schiedeanus* uniGenes code for soluble and extracellularly secreted enzymes; these are members of GH28 family and its modeled 3D structures resemble fungi endo-PG. The 3D structures of three of these *P. schiedeanus* enzymes (those which differ by only about 1 Å to fungi endo-PG used as reference) are shown in Figure 7 in which the conserved motifs, functional amino acid residues and even similarities regarding the electrostatic potential and topology of the active site are highlighted. These fungi endo-PG are actually inverting enzymes suggesting that the enzymes of the GH28 family do not conform to the standard inversion mechanism (Federici et al., 2001).

**FIGURE 8:**
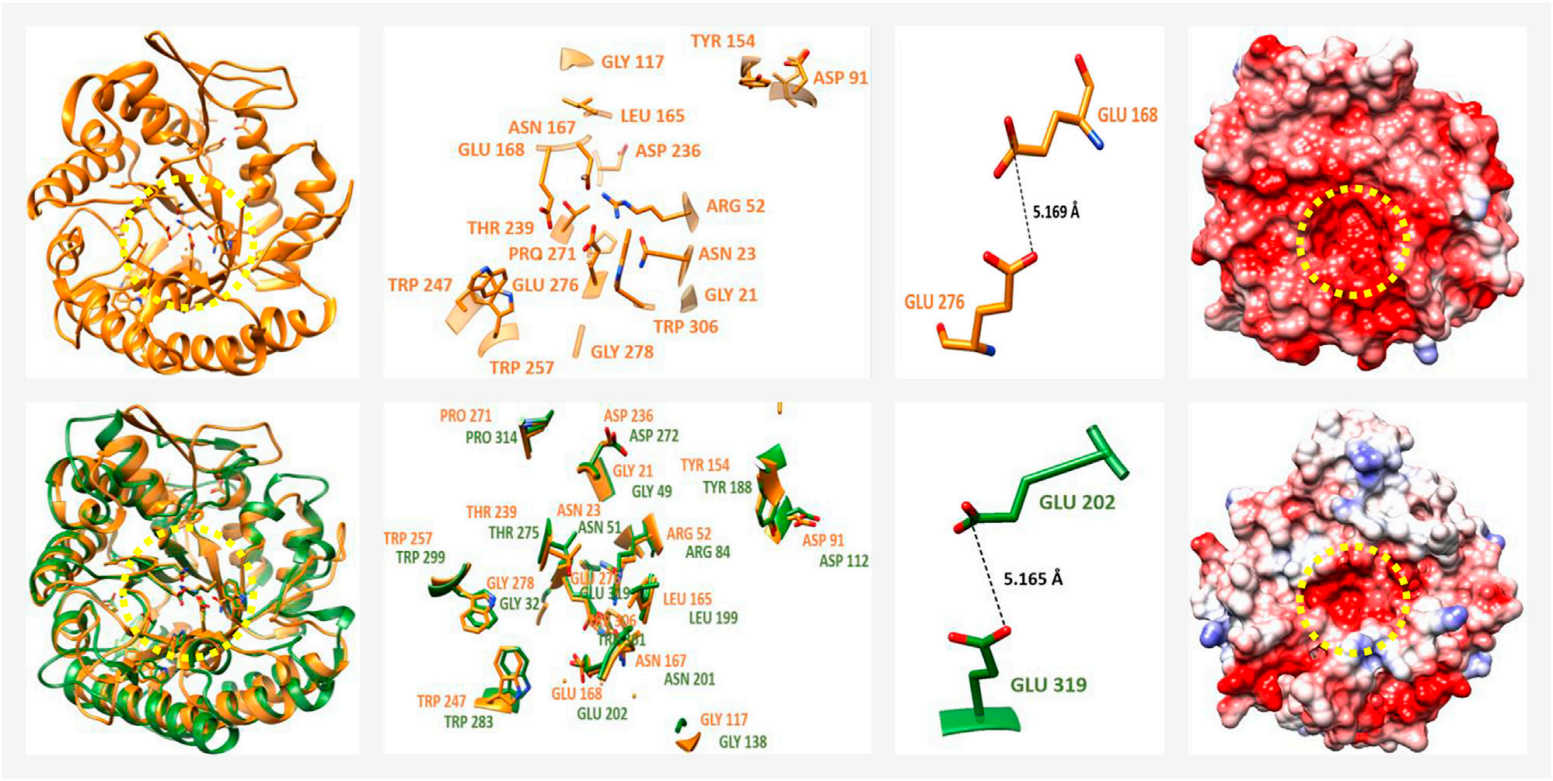
3D structure from an endo-β-1,4-mannanase (EβM-1,4) from *Psittacanthus schiedeanus* (enzyme member of GH5-7 family). From top to bottom, the EβM-1,4 from the phytopathogenic fungus *Aspergillus niger* BK01 (reference protein, PDB ID: 3WH9) followed by one of the modelled EβM-1,4 of the mistletoe *P. schiedeanus*. The EβM-1,4 from *P. schiedeanus* (UN045719, cyan) was superimposed with reference protein (red). From left to right (besides the 3D structures), a magnified image with the highly conserved amino acid residues which form the slot-like pocket from the active site is shown. This image is followed by another with the estimated distance between the catalytic residues (two glutamic acid (Glu) residues) and finally, an electrostatic surface charge representation. The yellow dotted circles show the active site with a relatively low presence of positive charges. After alignment and the superimposing of the 3D modelled structures, the equivalent position of every amino acid residue into the active site is shown. EβM-1,4 3D protein model from *P. schiedeanus* were constructed *in silico* based on the homology to cited proteins and their solved crystal structure.

Regarding the members of the GH family 5 (7-subfamily; GH5-7), a total of 13 *P. schiedeanus* uniGenes were identified (Supplementary Table S15). GH5-7 comprises mainly endo-β-1,4-mannanases (EβM-1,4), in which a total of 17 amino acid residues (G61, N63, R92, D131, G157, Y194, L205, N207, E208, D276, T279, W287, W297, P311, E316, G318 and W346) that are highly conserved in enzymes from phytopathogenic fungi (Sakon et al., 1996; Hilge et al., 1998; Dias et al., 2004) are also conserved in at least four soluble and extracellularly secreted *P. schiedeanus* enzymes (UN035485, UN039898, UN051984, and UN066323; Supplementary Figure S10). For the remaining *P. schiedeanus* enzymes the coding sequences obtained from the corresponding uniGenes are not complete, and thus not considered for downstream analysis. The homology percentage between mistletoe uniGenes and fungal reference enzymes ranges from 21.3 to 29.7% (Supplementary Table S18). The highly negative electrostatic potential and the topology of the active site (Sabini et al., 2000; Huang et al., 2014; Zhou et al., 2014), was confirmed with the modelled 3D structures (Figure 8). As previously observed with the PGs, the modelled EβM-1,4 mistletoe enzymes showed a RMSD of 1.5 A compared to at least 95% of the protein used as reference (beta-mannanase from *Aspergillus niger* BK01, PDB ID: 3WH9; Huang et al., 2014); Supplementary Table S19). Distance estimated between two catalytic residues is ∼5.5 Å (Figure 8), which strongly suggests that in these enzymes the hydrolysis occurs *via* a retention mechanism.

## Discussion

Transcriptomics allowed us to identify highly differentially expressed genes regulating seed germination, from seed inoculation to haustorium formation, in the photosynthetic hemiparasite *Psittacanthus schiedeanus*. This approach was complemented by detection of the enzymes and hydrolysis mechanism for breaking into the host. Our analysis provides a comprehensive view from possible ways by which mistletoes break through tissues of the host to haustorial formation.

### Transcriptional Basis for Haustorium Formation

Up until now, the biochemical and molecular processes occurring from seed deposition to haustorial formation and host penetration in Loranthaceae mistletoes had remained largely unknown. Most investigations on chloroplast genomes in parasitic and heterotrophic plants focused on non-photosynthetic species (Wicke et al., 2013; Petersen et al., 2015a, b; Cusimano and Wicke, 2016; Su and Hu, 2016; Su et al., 2021). Transcriptome sequencing has been specifically applied to reveal differentially expressed genes in the process of parasitism including those encoding hormones and proteins, and genes that induce/modulate haustoria formation and development in parasitic and non-parasitic Orobanchaceae species (Bandaranayake et al., 2010; Yang et al., 2015; Yoshida et al., 2019), shoot parasites (Convolvulaceae; *Cuscuta pentagona*, Ranjan et al., 2014; *Cuscuta campestris*, Vogel et al., 2018), mistletoes (*Taxillus chinensis*, Loranthaceae; Wei et al., 2020), and root hemiparasites in few plant families (e.g., *Santalum album*, Santalaceae, Zhang et al., 2012, 2015).

The assembled *P. schiedeanus* chloroplast whole genome showed a typical circularized quadripartite structure, very similar in size and structure to the chloroplast genomes of *Taxillus chinensis* and *T*. *stchuenensis* (Loranthaceae), two species with degenerated chloroplasts and restricted photosynthetic capacity (Li et al., 2017). A total of 105 genes, including 74 protein-coding genes, 27 transfer RNA genes, and four ribosomal RNA genes were identified, with approximately 95% of genome coverage when considering uniGenes identified as Cp-like sequences. *De novo* transcriptome assembly and analyses on *P. schiedeanus* data from several stages after seed inoculation allowed us the identification of genes associated with haustorium formation. Out of 140,467 uniGenes annotated using public databases, 2,000 were differentially expressed uniGenes throughout the initial stages of seed germination. At least one and up to seven conserved protein domains were identified in 88,611 uniGenes (63.08% from the total). As expected, the enriched GO-terms for the up-regulated uniGenes in *P*. *schiedeanus* comprise biological processes particularly linked to the development of a clearly photosynthetic anatomical structure (GO:0048856) that begins with seed germination. Specifically, responses to hormones (auxin and abscisic acid), temperature stimulus, water deprivation, and to oxidative stress are consistent with previous reports showing that the seed germination is induced commonly by the imbibition of water at a species-specific temperature and this imbibition of dry seeds activates a series of events (Bewley, 1997) including oxidation, degradation, and mobilization of accumulated reserve components (Penfield et al., 2005). Reactive oxygen species (ROS) also accumulate in seeds to a level that positively regulates seed germination (Leymarie et al., 2012). ROS are proposed to up-regulate abscisic acid (ABA) catabolism and promote gibberellic acid (GA) biosynthesis, thereby maintaining a dynamic balance between ABA and GA during seed germination (Liu et al., 2010). In addition, auxin signaling can negatively control seed germination through regulation of some transcription factors such as ABI3 (AT3G24650), a central regulator in ABA signaling and responsible for the transition between embryo maturation and early seedling development (Liu et al., 2013; Hussain et al., 2020). It should be noted that, the *P. schiedeanus* uniGene UN027634 is an ortholog to ABI3 (not a DEG but transcribed) which indicates that it is transcribed mainly in the mistletoe pre-sprouting seeds (T0).

### Expression Profiles and Signaling Pathways

The analysis of differential expression involved in development of polycotylous embryos indicated an increasing/decreasing expression pattern or a more dynamic differential expression. Six clusters were identified, with 2,096 differentially expressed uniGenes of two-fold or greater and a similar percentage of up- and downregulated uniGenes (53.9 and 65.3%, respectively). When DEGs were carefully analyzed, we noticed that a considerable number of these uniGenes were annotated in functional categories related to auxin and jasmonic acid (JA), differentially expressed and grouped particularly into expression clusters A and D, and orthologs/homologs of Amidase 1 and SUPERROOT 2. The auxin biosynthesis pathway is complex, its precursor (L-Trp), is synthesized in plastids and the subsequent steps take place in the cytosol. Four pathways for L-Trp-dependent auxin biosynthesis in higher plants are known (Ljung, 2013; Olatunji et al., 2017) (as reference, *see* Supplementary Figure S7), one of them (Indole-3-acetaldoxime or IAOx pathway) is unique to the Brassicaceae family (Sugawara et al., 2009). Most, but not all of the enzymes involved, have been functionally characterized (Olatunji et al., 2017). SUR2 is an enzyme that belongs to the cytochrome P450 family and it is involved in IAOx pathway; therefore, we suggest that, even when this uniGene showed a considerable percentage of similarity with SUR2 (∼40%) and was defined by OrthoMCL as a member of the orthoGroup941 (which also contain proteins from other species included in our analysis, see Supplementary Table S7), its involvement in the *P. schiedeanus* auxin biosynthesis pathway, should be discarded. This is consistent with subsequent analyses in which, after carrying out a search in *P. schiedeanus* uniGenes complete collection, we confirmed that no other uniGene was grouped in another OrthoMCL-defined protein family containing enzymes involved in the IAOx pathway (Supplementary Figure S7 and Supplementary Table S7).

Analysis of *P. schiedeanus* uniGenes indicated that those involved in auxin and JA signaling pathways were differentially expressed (Supplementary Figure S7 and Supplementary Tables S11, S12). Both auxins and JA participate in biological processes such as root development (Olatunji et al., 2017), lateral roots (LRs) formation (Du and Scheres, 2018; Santos Teixeira and Ten Tusscher, 2019), and adventitious roots (ARs) formation (Pacurar et al., 2014). Regarding LRs formation, while auxin promotes its formation, the JA functions as an inhibitor; in fact, it has been suggested that JA works like a selective counter-auxins in the lateral root formation process (Ishimaru et al., 2018). As known for other angiosperms, orthologs to these uniGenes are involved in LR or AR formation. This suggests that in *Psittacanthus* (and maybe in other rootless mistletoe species) orthologs to those uniGenes participate in the development of the intrusive organ to haustorial disk formation.

### Gene Regulatory Network in *Psittacanthus* Haustorium Formation

The haustorium formation in *P. schiedeanus* resembles, at least in part, the LRs or ARs formation processes. Genes associated with LR development have been previously linked to haustorium development in other lineages of parasitic plants (e.g., *Cuscuta australis*, Sun et al., 2018; *Striga asiatica*, Yoshida et al., 2019; and *Santalum album*, Zhang et al., 2015). Except for rootless plants, all plant species have a primary root derived from an embryonic radicle and different types of LRs. Most plants can develop ARs that display the same functions as LRs when developed from aerial tissues, mainly as an adaptive response to stress such as wounding or flooding. LRs and ARs develop post embryonically and share key elements of the genetic and hormonal regulatory networks but are subject to different regulatory mechanisms (Atkinson et al., 2014; Bellini et al., 2014). In addition, LRs and ARs develop from different tissues and consequently from different cell types. We identified a complex gene regulatory network that might be involved in *P*. *schiedeanus* haustorium formation, highly similar to the early steps of ARs and LRs formation, which require and depend on gene networks regulated mainly by auxin, but also by jasmonic acid (JA) and strigolactones (Figure 6D and Supplementary Figure S7).

Despite similarities between AR and LR morphogenesis, their regulation exhibits some clear differences (summarized in Bellini et al., 2014). The most obvious overlap is the central role of auxin signaling controlling initiation as well as subsequent primordia development and emergence. Auxin response factors such as ARF7 (Moreno-Risueno et al., 2010), ARF17 (Gutierrez et al., 2009), ARF6, ARF8 and ARF19 (De Rybel et al., 2010), and some auxin repressor proteins INDOLE-3-ACETIC ACID INDUCIBLE28 (IAA28; as well as IAA8 and IAA19 (Rogg et al., 2001; De Rybel et al., 2010) have been functionally characterized in *A. thaliana* and some other plant species, and its involvement in both, LRs and ARs primordium formation is known. First, IAA28, ARF7, and ARF19 control the expression of downstream target genes such as some GATA transcription factors (De Rybel et al., 2010). These regulators have a role in pre-branch site formation, which occurs when the auxin response oscillation has reached a maximum in this region, demarking the position of the future lateral root primordium (De Rybel et al., 2010). Initiation and patterning are regulated by the expression of ARF6, ARF8, and ARF17 which are controlled by MIR167 and MIR160, respectively, and oppositely regulate jasmonic acid homeostasis *via* regulating jasmonic acid-modifying GRETCHEN HAGEN3 (GH3) enzymes (Gutierrez et al., 2009, 2012; Atkinson et al., 2014; Bellini et al., 2014). Additional hormone signals are known to be involved in ARs formation (Bellini et al., 2014). For example, strigolactones, whose biosynthesis is orchestrated by the MAX proteins (Nelson et al., 2011; Kumar et al., 2019), block ARs formation in *A. thaliana*, most likely by interfering with auxin transport (Kohlen et al., 2012; Rasmussen et al., 2012; Atkinson et al., 2014; Bellini et al., 2014). Notice that auxin transporters both, influx and efflux (LAX3 and PIN1, respectively), are required to control the auxin accumulation in the early stages of ARs organogenesis (Della Rovere et al., 2013); a localized increase in auxin levels subsequently enables the activation of expansins and cell wall remodeling enzymes (Santos Teixeira and Ten Tusscher, 2019). PLT genes were demonstrated to control the proper expression of some PIN auxin transporters, as well as the WOX5 transcription factor which is involved in the specification and maintenance of the stem cells (QC cells) in the roots’ apical meristem genes (Santos Teixeira and Ten Tusscher, 2019).

*Psittacanthus*, is one of the most species-rich genera of Loranthaceae (Santalales) and until now, the least studied regarding molecular mechanisms involved on seed germination and ontogeny, differentiation, and haustorium formation. Also, it is one of the two genera of Loranthaceae (besides *Aetanthus*) described as lacking endosperm and with polycotylous embryos (Kuijt, 1970, 2009; Kuijt and Hansen, 2015; Ornelas et al., 2022). The veracity of these atypical phenotypic traits was questioned by González and Pabón-Mora (2017). However, the subsequent rebuttal questioned the correct identification and interpretation of plant materials used by these authors (Kuijt, 2018). According to Kuijt’s descriptions (Kuijt, 1970, 2009; Kuijt and Hansen, 2015), the bulk of the *Psittacanthus* seed consists of fleshy, three-sided cotyledons that develop rapidly once the seeds are cemented on the host branch through the viscous material which surround them (viscin). Its basal region is darkened after 1 day of inoculation and includes the suspensor that is pushed aside by the emerging haustorial organ. These intrusive organs are not morphologically terminal but emerge laterally from the flanks of the apical dome close but not from the root-like apical meristem (Figures 6A–C and Supplementary Figure S8; Kuijt, 1970, 2009). These descriptions correspond to our observations on polycotylous embryos whose development was analyzed at 7, 14, 21 and 28 dag (Figure 1). In our experiments, we also noticed that 28 days after seed inoculation on wooden rectangle sticks, cotyledons start to lose turgor and after a period, perished. In contrast, if the seeds are inoculated on branches of a live host, after 35–40 days, the first true leaves started to emerge (Figure 1L). Together, our results suggest that the intrusive organs develop during haustorium formation in a similar way to ARs and/or LRs given the considerable number of DEGs identified as orthologs to those genes involved in the ARs and LRs formation. Considering that intrusive organs are not always successful in invading the host’s tissues, this process of regeneration/formation of intrusive organs is repeated before the haustorial organ is securely established.

### Enzymatic Hydrolysis Mechanisms

Numerous pathogenic or parasitic organisms attack plants to obtain nutrients from them, parasitic fungi are without a doubt a good example, but so are parasitic plants and the plant-parasitic nematodes. While it is true that these organisms are distinct types of parasites, they share some common features on their strategies for breaking into their host. The first challenge for these pathogens is to breach the host plant cell wall, which is the protecting physical barrier against attack (Underwood, 2012). To penetrate and break down this barrier, most phytopathogenic fungi (and oomycetes) have developed an arsenal of tools such as the secretion of cell wall-degrading enzymes (CWDEs) including pectinases, polygalacturonases, glucanases, cellulases, and xyloglucanases (Nühse, 2012). For its part, plant-parasitic nematodes breach the plant cell walls by protruding a sclerotized stylet from which CWDEs are secreted (Mitsumasu et al., 2015). A crucial difference between the growth of fungal and parasitic plant haustoria is that haustorium produced by fungi actually grows within host cells, whereas in parasitic plants cause ruptures to the cell wall of host tissues but does not penetrate host cells except in cases in which parasitic plant haustorium cells invade host vessels *via* pit apertures without cell wall rupture (Mitsumasu et al., 2015). Regarding parasitic plants, host penetration is poorly studied but it has been reported that species in *Striga* and *Orobanche*, two genera of root obligate parasitic angiosperms, cell wall degrading enzymes are highly expressed during haustorium formation (Mitsumasu et al., 2015). In *Cuscuta reflexa*, high pectinolytic activity in haustorial extracts and high expression levels of pectate lyase genes suggest that the parasite contributes directly to wall remodeling during host plant penetration (Johnsen et al., 2015; Olsen et al., 2016). It is also possible that parasitism in some mistletoe species is assisted by endophytic fungi, which can secrete cellulases and assist the mistletoe’s haustorium to break through the cell walls as well as intercellular space tissues of the host (Ding et al., 2008).

The modeled 3D structures of the enzymes from *P. schiedeanus* provided some evidence about their action mechanism, that is, how these enzymes carry out the hydrolysis of the glycosidic bond. This enzymatic reaction takes place *via* two major mechanisms giving rise to either an overall retention, or an inversion of anomeric configuration (McCarter and Withers, 1994; Davies and Henrissat, 1995). At least two catalytic residues (acid residues) make possible the hydrolysis in the active site and as previously reported, an average distance between these residues is a key factor (∼5.5 Å is the typical distance for the retaining enzymes while ∼10 Å is the distance required in inverting enzymes; McCarter and Withers, 1994). The estimated distance between catalytic residues in *P. schiedeanus* modelled enzymes ranges between 3.893 and 6.589 Å (Figure 7), which suggests that the hydrolysis occur *via* the retention mechanism. However, the distances observed between catalytic residues are highly similar to the distances reported for the endo-PG of *Fusarium moniliforme* (enzyme used as reference in the superposition analysis) and for some other phytopathogenic fungi endo-PG enzymes as well (van Santen et al., 1999; Federici et al., 2001). These fungi endo-PG are actually inverting enzymes suggesting that the enzymes of the GH-28 family do not conform to the standard inversion mechanism (Federici et al., 2001). However, amino acid residues that are highly conserved in enzymes from phytopathogenic fungi (Sakon et al., 1996; Hilge et al., 1998; Dias et al., 2004) are also conserved in soluble and extracellularly secreted *P. schiedeanus* mistletoe enzymes, and distance estimated between catalytic residues strongly suggests that in these enzymes the hydrolysis occur *via* a retention mechanism.

Undoubtedly, the development and function of the haustorium remain surrounded by many questions. Considering its basic function, which is attachment to a substrate and water and nutrient uptake, parallels obviously occur between roots and haustoria. In *Cuscuta* species, haustoria originate not from the roots, but from twining stems, and in this case, the haustorium is generally interpreted as a modified and reduced adventitious root (Yoshida et al., 2016; Kokla and Melnyk, 2018). Our results suggest that something similar occurs in *Psittacanthus* species in which the intrusive tissues leading to haustorial formation emerge laterally from the dome formed in the basal region of the star-shaped bodies (vegetative organs with high content of transcriptionally active plastids). It is possible that the scar-like dark area which also includes suspensor remnants and is pushed aside by the emerging haustorial organ also deter the development of the apical meristem that belongs to the embryo and that would give rise to a radicle-like organ (primary root). This explains why the formation of intrusive tissues that give rise to the haustorium share many molecular aspects with adventitious root formation, the foregoing, at least regarding three key aspects like cell division, primordium formation, and organization of apical meristem (Figure 6D and Supplementary Tables S9, S13, S14).

Despite similarities between haustoria and roots, it is unquestionable the large physiological and anatomical disparities that encloses the haustorium itself. In fact, some transcriptome analyses performed in a few parasitic plants suggest that, during evolution, haustoria might have co-opted genes normally expressed in roots, but also in floral tissue. In root parasites of Orobanchaceae (*Triphysaria versicolor*, *Striga hermonthica*, and *Phelipanche aegyptiaca*), molecular evidence indicates that they evolutionarily recruited many genes for haustorial development and host penetration from genes that were involved in other processes in related non-parasitic plants, primarily root (Sun et al., 2018) or flower development, but with some genes co-opted from other tissues (Yang et al., 2015; Yoshida et al., 2016). These putative parasitism genes are also upregulated in the haustoria of *Cuscuta campestris* (Ranjan et al., 2014), and auxin-mediated regulation of haustorium initiation shared by both root and stem parasitic plants (Yoshida et al., 2016; Jhu et al., 2021, 2022), would support the hypothesis that stem parasites also co-opted the root parasite program into haustorium development. Based on this, some authors proposed that haustoria might be interpreted as morphological misfits (Rutishauser and Isler, 2001; Teixeira-Costa, 2021a). Teixeira-Costa has further reviewed various lines of evidence including her comparison of the haustorium morphology, ontogeny, and anatomy across all 12 different clades that include parasitic plants and suggested that the haustorium cannot be considered as fully homologous to neither roots, nor stems. In fact, she proposed that this parasitic plant organ would be best interpreted as a “root-shoot mosaic” (Teixeira-Costa, 2021a, b). According to our results, we suggest that the early stages of intrusive organs formation in some parasitic plants would be highly similar to those that give place to adventitious roots formation. However, it is also possible that once they successfully establish contact with the host xylem, haustoria, at least in terms of their functions, might resemble both organs, that is, roots and stems. This could be particularly relevant in endophytic parasitic plants belonging to group 1 (Santalaceae species). As specified by Teixeira-Costa et al. (2021), this species group shows a dramatically different developmental pattern, featuring early cell differentiation and tissue organization, and little effect on host anatomy and cambial activity, while species from Apodanthaceae, Cytinaceae, Mitrastemonaceae, and Rafflesiaceae families (group 2), show a common developmental pattern characterized by late cell differentiation (Teixeira-Costa et al., 2021).

## Data Availability

The datasets presented in this study can be found in online repositories. The names of the repository/repositories and accession number(s) can be found below: https://www.ncbi.nlm.nih.gov/genbank/, PRJNA803466, https://www.ncbi.nlm.nih.gov/genbank/, GJSQ00000000, https://www.ncbi.nlm.nih.gov/genbank/, OM677836.
