# Supplementary material for: Transcriptional Basis for Haustorium Formation and Host Establishment in Hemiparasitic Psittacanthus schiedeanus Mistletoes
Source: Front Genet. 2022 Jun 13;13:929490. doi: 10.3389/fgene.2022.929490 (PMC9235361; doi:10.3389/fgene.2022.929490)
Supplement: Supplementary file 3 [file DataSheet1.pdf]

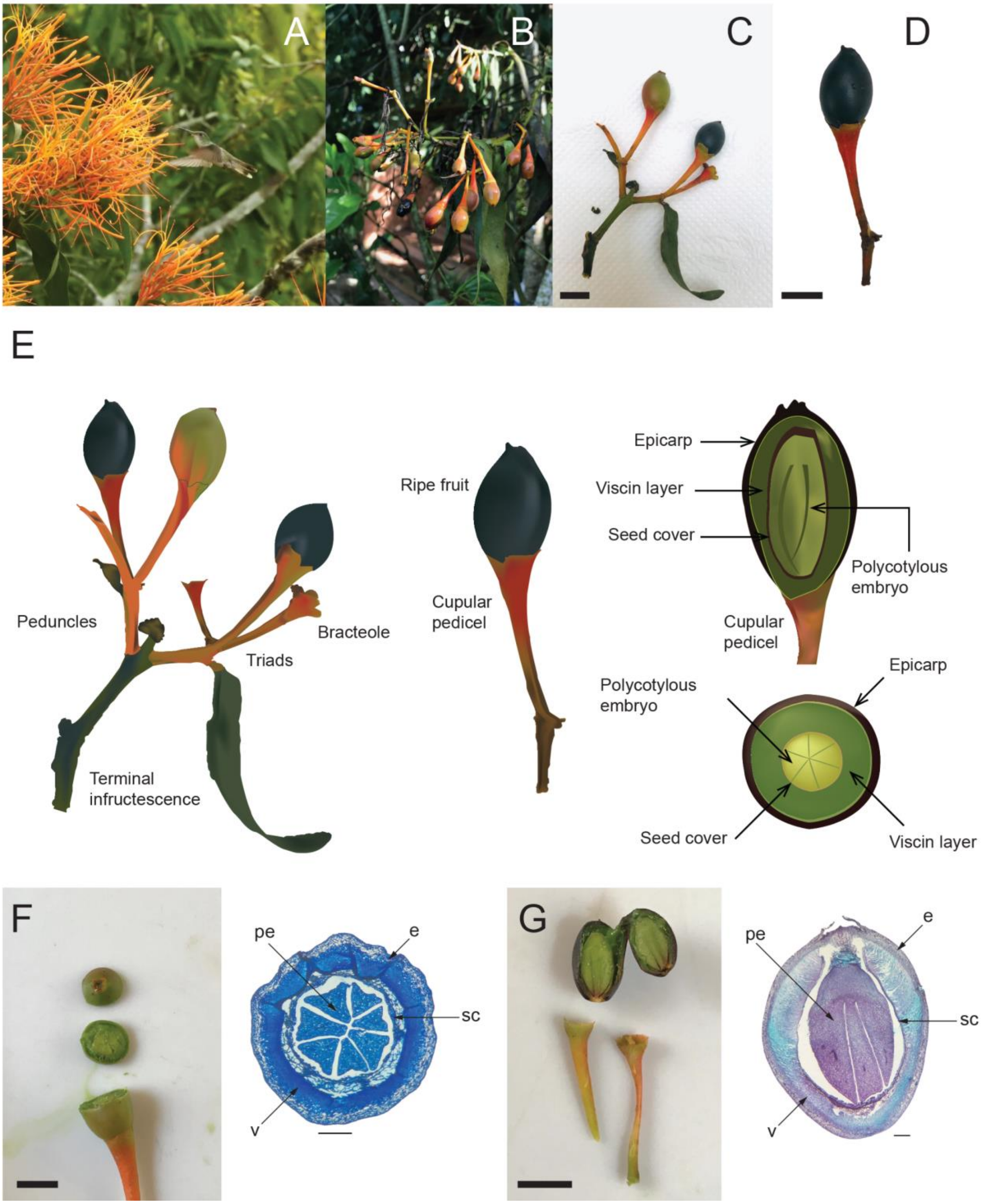

Supplementary Figure S1. Anatomy and morphology of *Psittacanthus schiedeanus* fruits. **(A)** Inflorescence and **(B)** infructescence of hummingbird-pollinated *P. schiedeanus*. **(C)** Treads of fruits at various developmental stages. Note fruit color changes from green when immature to purplish-black when ripe **(D)**. **(E)** Illustration of infructescence and fruit morphology and anatomy based on photographs. **(F)** Cross-section of developing fruit stained with toluidine blue. **(G)** Longitudinal section of a ripe fruit developing fruit stained with safranin and fast green FCF. Note the inverted position of the polycotylous embryo. Photo by Juan Francisco Ornelas **(A–D** and **F–G)**. Scale bar = 0.5 cm. Illustrations by Julieta Ornelas Peresbarbosa **(E)**. Preparation and microphotographs of fruit sections: Sonia Galicia. e = epicarp, pe = polycotylous embryo, sc = seed cover, v = viscin layer. Scale bar = 1 mm (see also Ornelas *et al.*, 2022).

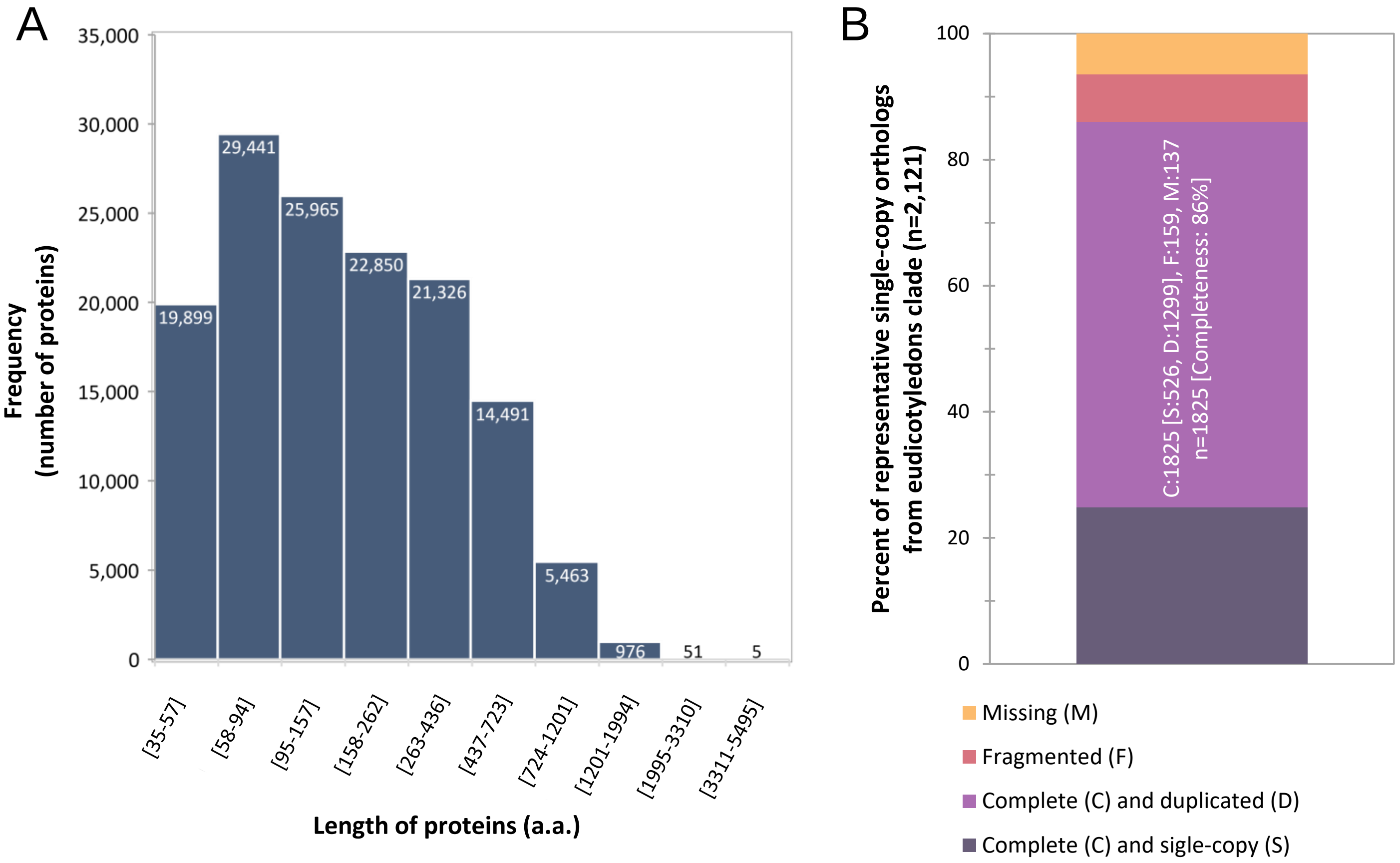

Supplementary Figure S2. Histogram of the proteins' length and the completeness of *Psittacanthus shiedeanus* transcriptome. **(A)** Histogram showing the distribution of lengths of proteins/peptides translated from coding regions identified into the *P. schiedeanus* uniGenes. x-axis is the length of proteins (amino acids; a.a.) and y-axis the number of proteins at each length range. **(B)** Completeness estimated based on single copy orthologs shared between flowering plants belonging to eudicotyledons clade (n = 2,121). Colors in the bar represent the different classes resulting of the BUSCO assessment.

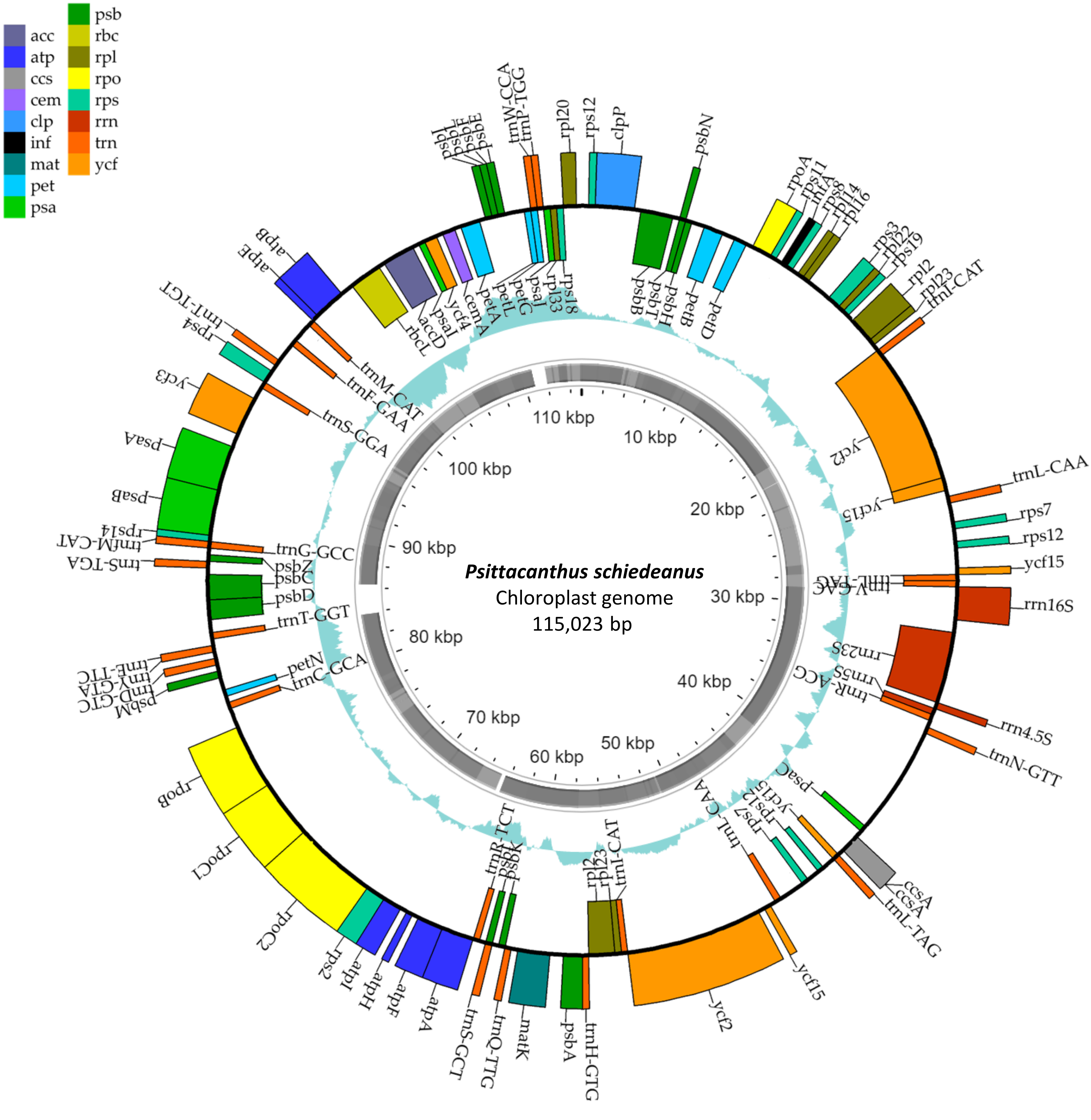

Supplementary Figure S3. Chloroplast genome map of *Psittacanthus schiedeanus* species. Genes drawn inside the circle are transcribed clockwise, whereas those outsides are counterclockwise. Different gene functional groups are color coded. The circles inside of chloroplast genome map show in cyan the GC-content (intermediate ring) and the coverage (blocks grey at the innermost ring) which is provided by sequences identified as Cp-like uniGenes. The genome map was drawn using CpGAVA (Liu *et al.*, 2012).

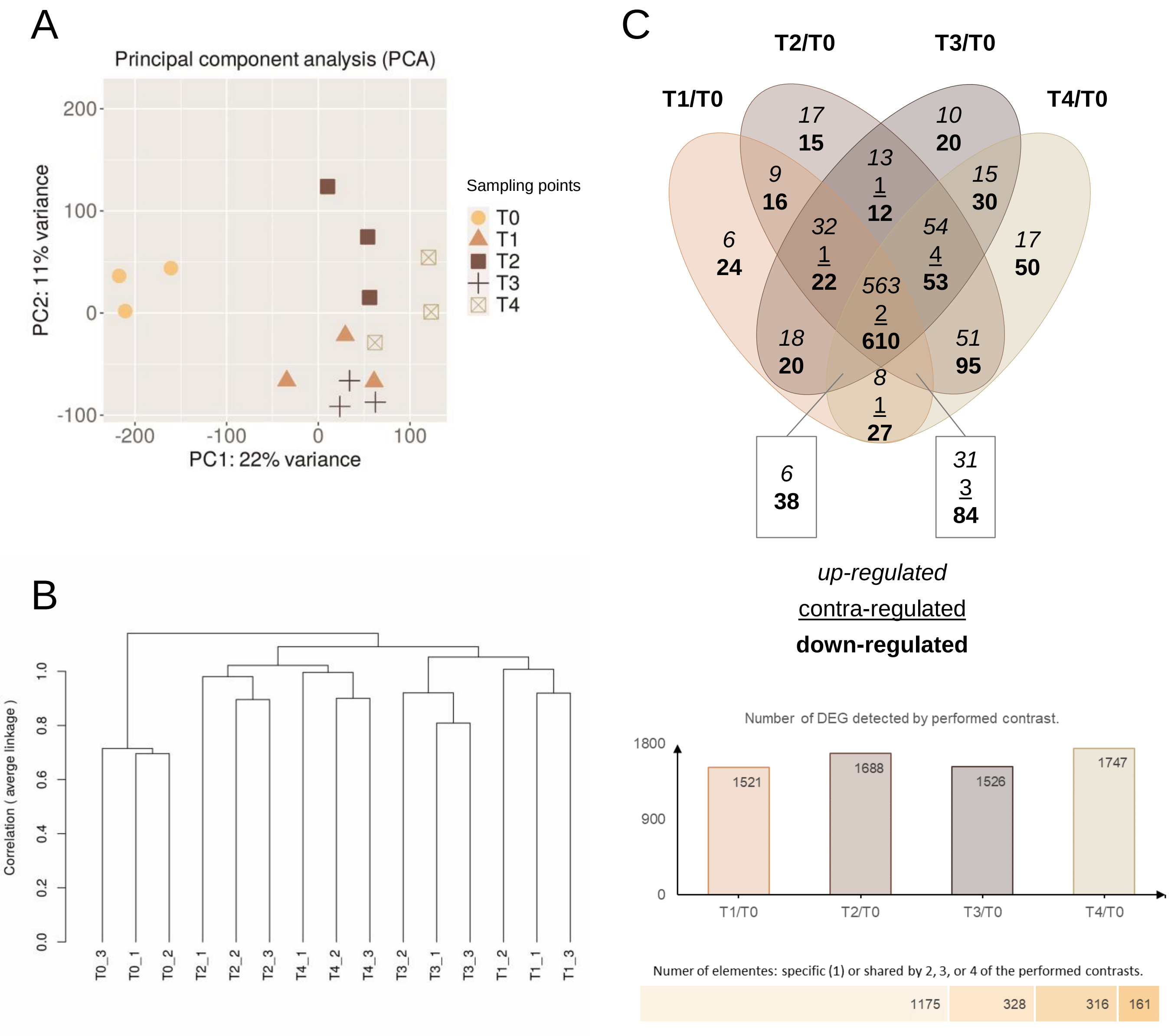

Supplementary Figure S4. Gene expression profile changes of *Psittacanthus schiedeanus* before and after experimental inoculation. **(A)** Principal component analysis (PCA) plot displaying all 15 generated and used libraries in the presented study [T0: *P. shiedeanus* pre-sprouting seeds; T1 – T4: sampling points at 7, 14, 21, and 28 days after germination (dag); three independent biological replicates of each]. PC1 (x-axis) and PC2 (y-axis), describe 22% and 11% of the variability, respectively. PC analysis was applied to transcripts per million (TPM) values. **(B)** Hierarchical clustering tree of sampling points (and their independent biological replicates) performed with the maximum expression level values (TPM) at the top 75%. The number followed by the abbreviations of sampling points indicates the replicates. The correlation (average linkage) for the clades is shown at the left of the tree. **(C)** Venn diagram from differentially expressed uniGenes (DEG) which were identified by samples pair-wise comparisons as follow: T1/T0, T2/T0, T3/T0 and T4/T0.

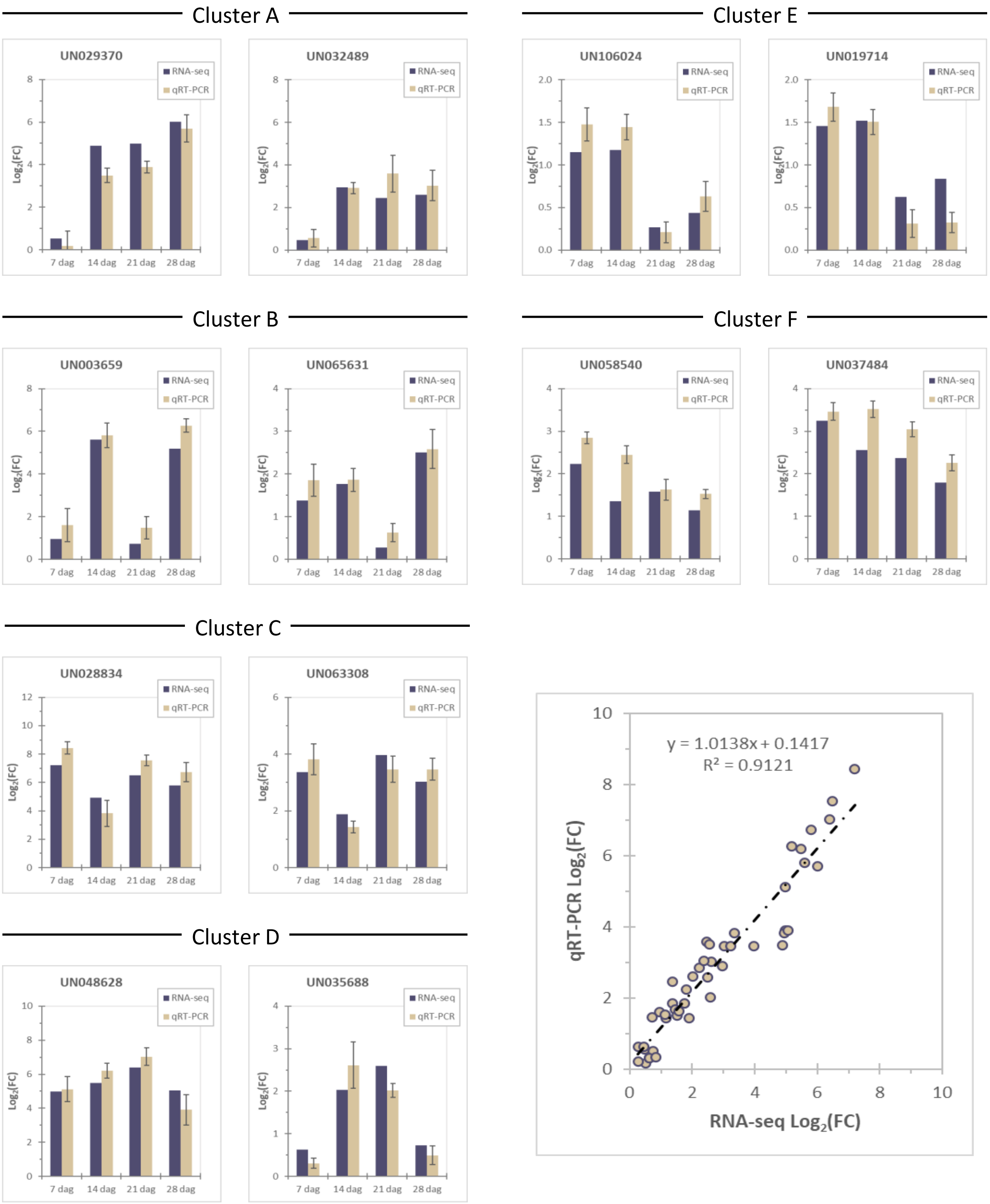

Supplementary Figure S5. Validation of RNA-seq results using real-time PCR (qRT-PCR). Bar plots were generated from the ratio of transcripts levels obtained by qRT-PCR ($2^{-\Delta\Delta CT}$ relative expression level, beige bars), and compared with the expression profile obtained by RNA-seq (Log_2FC, purple bars). The qRT-PCR data were obtained by analyzing the transcript level of different uniGenes in the star-shaped chlorophyllous bodies development at 7, 14, 21, and 28 days, which were normalized using the transcript level obtained from pre-sprouting seeds. The Scatter plot shows simple linear regression and the R-squared (R^2) between relative expression obtained by qRT-PCR (y-axis) and Log_2FC values (x-axis) obtained by RNA-seq. Two uniGenes belonging to each of the six distinct clusters formed according to their expression profiles (Figure 3 and Supplementary Table S9) were randomly selected to their validation.

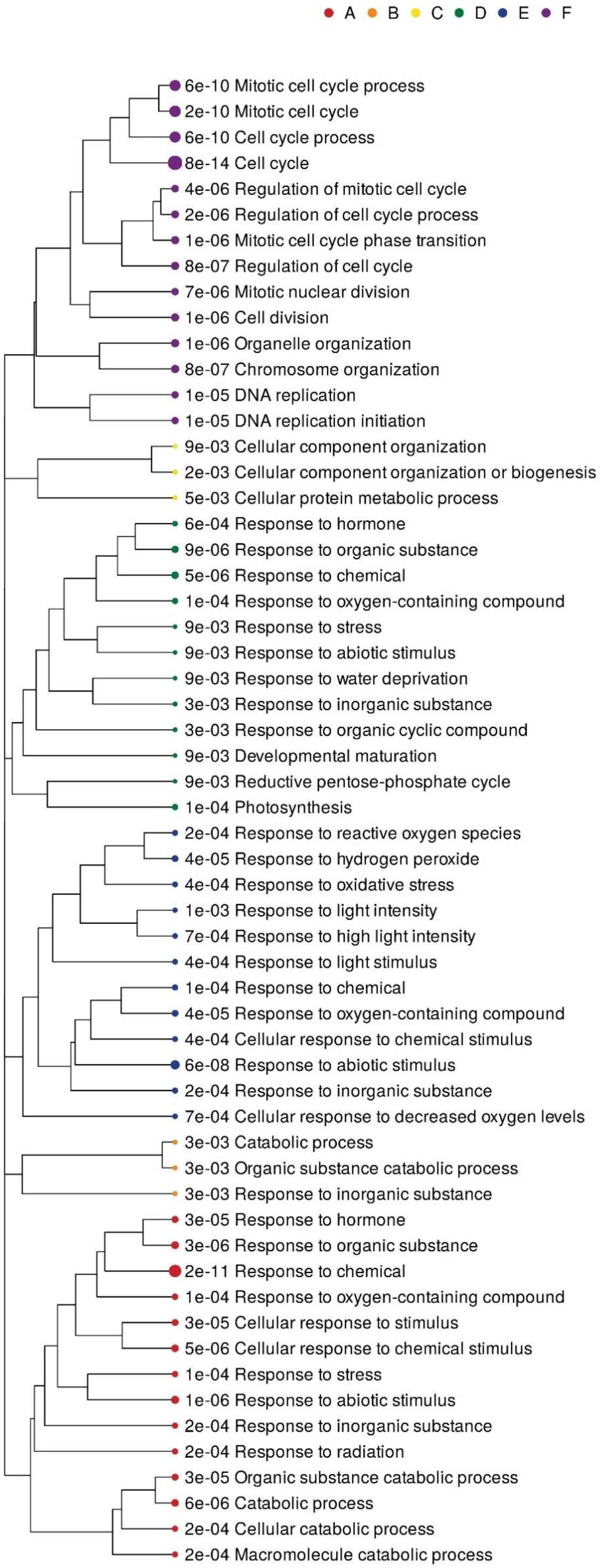

Supplementary Figure S6. Functional enrichment analysis of differentially expressed *Psittacanthus schiedeanus* uniGenes. Tree view shows the relationship among GO-terms enriched (Biological Process) on each of the six lists of DEGs which were defined based on the similarity of their expression profile (Figure 3 and Supplementary Table S9). Gene sets closer on the tree share more genes. Sizes of dot correspond to adjusted *p*-values. Dots in the tree were colored according to its membership to the cluster (from A to F).

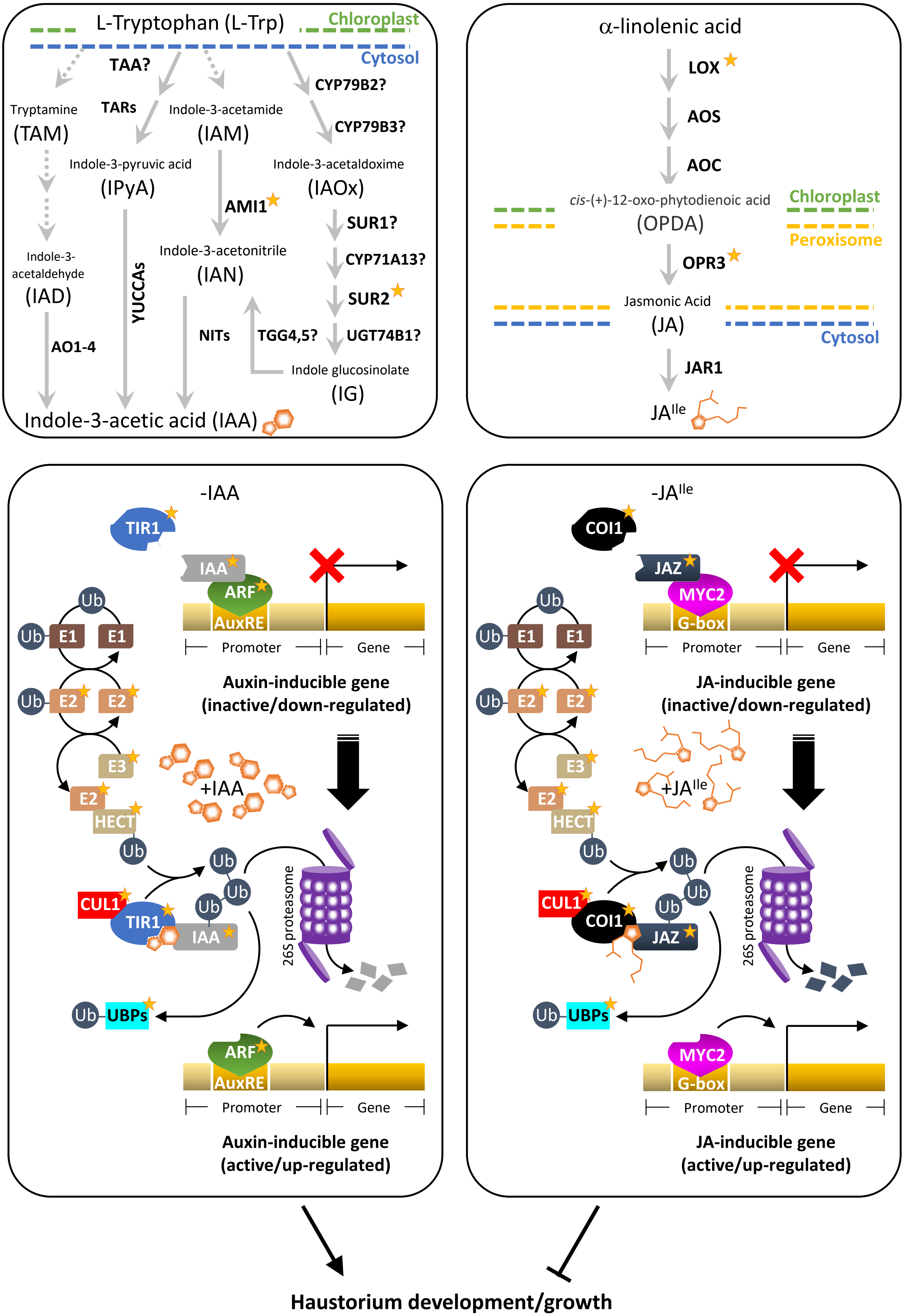

L-Tryptophan (L-Trp)
Chloroplast
Cytosol
TAA?
TARs
CYP79B2?
CYP79B3?
Tryptamine
(TAM)
Indole-3-acetamide
(IAM)
Indole-3-pyruvic acid
(IPyA)
Indole-3-acetaldoxime
(IAOx)
AMI1
SUR1?
CYP71A13?
SUR2
UGT74B1?
Indole-3-acetaldehyde
(IAD)
YUCCAs
Indole-3-acetonitrile
(IAN)
NITs
TGG4,5?
Indole glucosinolate
(IG)
AO1-4
Indole-3-acetic acid (IAA)
α-linolenic acid
LOX
AOS
AOC
cis-(+)-12-oxo-phytodienoic acid
(OPDA)
Chloroplast
Peroxisome
OPR3
Jasmonic Acid
(JA)
Cytosol
JAR1
JA-Ile
-IAA
TIR1
IAA
ARF
AuxRE
Ub
E1
E2
E3
HECT
Promoter
Gene
Auxin-inducible gene
(inactive/down-regulated)
+IAA
CUL1
26S proteasome
UBPs
Auxin-inducible gene
(active/up-regulated)
-JA-Ile
COI1
JAZ
MYC2
G-box
JA-inducible gene
(inactive/down-regulated)
+JA-Ile
JA-inducible gene
(active/up-regulated)
Haustorium development/growth

Figure S7. *Psittacanthus schiedeanus* uniGenes involved in auxin (IAA) and jasmonic acid (JA) biosynthesis and its signaling pathways. Right to left, boxes with the IAA- and JA-relative genes, respectively. From top to bottom, synthesis and signaling pathways schematic representations are shown. The four know pathways for L-Trp-dependent IAA biosynthesis in higher plants are shown: the IAOx, IAM, IPyA, and TRA pathways. The biosynthesis of IAA precursor, L-Trp, takes place in plastids (Chloroplast). The following steps to be located in the cytosol. On the other hand, the synthesis of JA which includes its active amino acid-conjugates, the JA-Ile. Organelles involved in JA biosynthesis are chloroplast and peroxisome. The enzymes known to operate in each biosynthesis pathway are shown next to gray arrows. Dashed gray arrows represent enzymatic reactions with still unknown catalyzing enzymes. Regarding signaling pathways, the canonical representation is shown. At low auxin (or JA) concentrations, IAA (or JAZ) proteins repress the activity of ARFs (or MYCs) TFs while at high auxin (or JA) concentrations, IAA (or JAZ) proteins be bound by a co-receptor complex (TIR1/CUL1-Aux/IAA or COI1/CUL1-JA/JAZ, as the case may be), which triggers ubiquitination and subsequent degradation of the repressor (IAA/JAZ proteins) via the proteasome thereby enabling ARF (or MYC) TFs activity. The ubiquitination requires the coordinated activity of Ub-activating (E1), Ub-conjugating (E2), and Ub-ligating enzymes (E3/HECT) for Ub attachment to substrate protein (IAA or JAZ, as the case may be). Ubiquitination can be also reversed through the action of deubiquitinating enzymes such ubiquitin-specific proteases (UBPs). The yellow star next to the name of some enzymes indicates that they were identified as DEGs. Notice that, with a few exceptions (the enzymes with a question mark), we identified in the uniGenes collection generated in the present study, *P. schiedeanus* homologs/orthologs from all proteins/enzymes represented in the figure. Next, abbreviations (and *P. schiedeanus* uniGenes identifiers). TAA1: Tryptophan aminotransferase; TAR: Tryptophan aminotransferase related (UN016993, UN037752, UN018813, UN034662, UN030980); YUCCAs: yucca genes encoding flavin-containing monooxygenases (UN055366, UN057815, UN088982); AMI1: Amidase 1 (UN018669); NIT: Nitrilase (UN048885, UN056006, UN051288, UN056262); SUPERROOT 2 (SUR2): enzyme belongs to cytochrome P450 family (UN042299); UGT74B1: UDP-glucose:thiohydroximate S-glucosyltransferase (UN065476); TGG-4 & -5: myrosinases; LOX: Lipoxigenase (UN015550, UN043638), AOS: Allen oxide synthase (UN033883, UN037900); AOC: Allen oxide cyclase (UN096822, UN114400); OPR3: Oxophytodienoate-reductase 3 (UN061490, UN018008); JAR1: Jasmonate-amido synthetase (UN030079, UN039880); TIR1: Auxin receptor that mediates auxin-regulated transcription (UN029761, UN005258); CUL1: Cullin (UN045078, UN114279); UBPs: ubiquitin-specific proteases (UN026586, UN044388, UN038949); COI1: JA receptor that mediates JA-regulated transcription (UN016743); JAZ: Jasmonate-zim-domain protein (UN045403); MYCs: are transcriptional activator; in particular, MYC2, is a positive regulator of lateral root formation (Kazan and Manners, 2013); UN011994, UN012170); E1: Ub-activating enzymes (UN001819, UN002964, UN004669, UN004726, UN004781, UN004890, UN006460, UN006609, UN007953, UN008143, UN052464); E2: Ub-conjugating enzymes (UN034684, UN142964, UN099451); E3 and HECT: Ub-ligating enzymes (UN000514, and UN063918, respectively); UBPs: ubiquitin-specific proteases (UN023784, UN065358, UN012763, UN044388, UN005449, UN026586, UN038949). *P. schiedeanus* ARF and IAA transcriptions factors identified like DEGs are shown in Supplementary Tables S11-S14 and Figure 6.

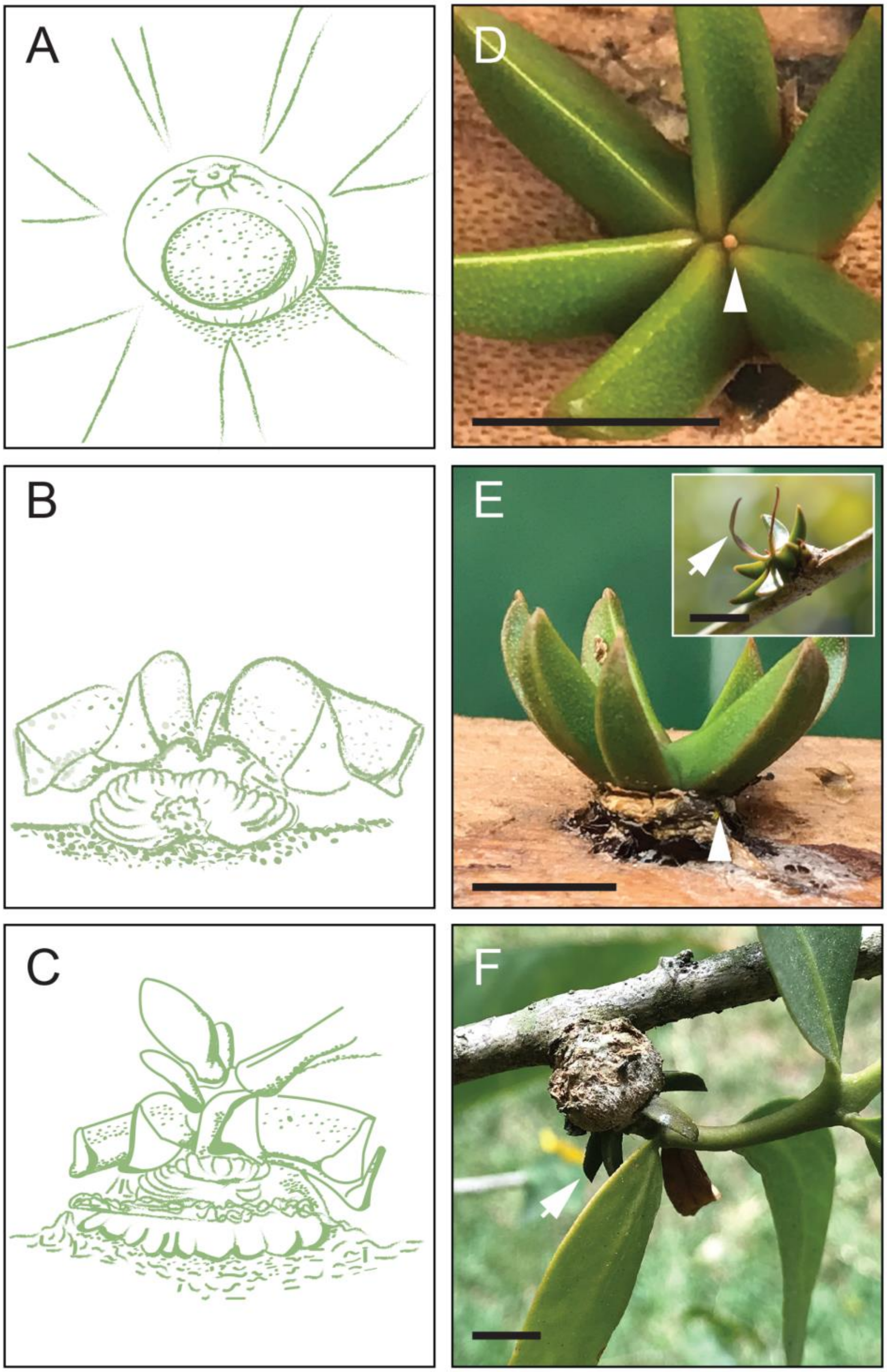

Supplementary Figure S8. Development of star-shaped chlorophylous bodies (seedlings) of *Psittacanthus schiedeanus* species. **(A)** Seedling inverted which shows the emergence of the (oval) primary haustorium just below the small suspensor scar. **(B)** An older seedling shows the grooved haustorial cushion formed upon penetration. **(C)** Established seedling with early primary leaves. The tips of prismatic lobes of star-shaped bodies are removed in **(B)** and **(C)**. (Re-drawn from Kuijt, 1970, 2009; Drawings by Julieta Ornelas Peresbarbosa). **(D)** The seedling from above with an apical shoot growing from the polycotylous embryo. **(E)** Older seedling showing the grooved haustorial cushion formed upon penetration and the emergence of true foliar leaves from the middle of the cotyledons (inset). **(F)** Established seedling with grooved haustorial cushion and the primary shoot emerging from the middle of the cotyledons, which remain for several months centre. Photos by Juan Francisco Ornelas (**D–F**) and Eliezer Cocoletzi (inset). Scale bar = 0.5 cm.

```
             ....|....| ....|....| ....|....| ....|....| ....|....| ....|....| ....|....|
                   215        225        235        245        255        265        275
EnPoGaAn     VTINNADGD- TQ-------G GHNTDAFDVG NSVGVNIIKP WVHNQDDCLA VNSGE-NIWF TGGTCIGGHG
EnPoGaAp     VTIDNSAG-- TA-------E GHNTDAFDIG SSTYINIDGA TVYNQDDCLA INSGS-HITF TNGYCDGGHG
EnPoGaAo     VTIDNSAG-- TA-------E GHNTDAFDVG SSTYINIDGA TVYNQDDCLA INSGS-HITF TNGYCDGGHG
EnPoGaAa     ITIDNSDGD- DN-------G GHNTDAFDIG TSTYVTISGA TVYNQDDCVA VNSGE-NIYF SGGYCSGGHG
EnPoGAFm     LILDNRAGDK PNAKSGSLPA AHNTDGFDIS SSDHVTLDNN HVYNQDDCVA VTSGT-NIVV SNMYCSGGHG
UN027741     LEVYAPA--- ---------I SPNTDGIHIS ASTRVEVKDS IVRTGDDCIS IVSNSSAIKI KNIVCGPGHG
UN029718     LEVYAPA--- ---------I SPNTDGIHIS ASTRVEVKDS IVRTGDDCIS IVSNSSAIKI KNIVCGPGHG
UN040504     LEVYAPA--- ---------I SPNTDGIHIS ASTRVEVKDS IVRTGDDCIS IVSNSSAIKI KNIVCGPGHG
UN045719     LEVYAPA--- ---------I SPNTDGIHIS ASTRVEVKDS IVRTGDDCIS IVSNSSAIKI KNIVCGPGHG
UN048617     LEVYAPA--- ---------I SPNTDGIHIS ASTRVEVKDS IVRTGDDCIS IVSNSSAIKI KNIVCGPGHG
UN062967     LRIIAPE--- ---------K SPNTDGIDIE SSGNIRILDS FIGTGDDCIA IGGGSYNIMV SGISCGPGHG
UN068437     LRIIAPE--- ---------K SPNTDGIDIE SSGNIRILDS FIGTGDDCIA IGGGSYNIMV SGISCGPGHG
UN070596     VRVTAAG--- ---------D SPNTDGIHVQ TSTHVSIVSS TIGTGDDCVS IGPGTSNLWM ENIGCGPGHG
UN071326     ITIDNTAGDV TN-------G GHNTDAFDVG SSTGITISNA NIKNQDDCLA VNSGK-NIHF TGGTCSGGHG
UN093239     VRVTAAG--- ---------D SPNTDGIHVQ TSTHVSIVSS TIGTGDDCVS IGPGTSNLWM ENIGCGPGHG
UN097465     ITIDNSAGDK TN-------G GHNTDAFDVG SSTGITISNA NIKNQDDCLA VNSGK-NIHF TGGTCSGGHG
UN101273     LKVSAAS--- ---------N SPNTDGIHIT GSTNVVVRDT IIGTGDDCIS IVNASSNIMV KRVVCGPGHG
UN153232     VRILAHD--- ---------E SPNTDGIRIG RSNHISISRS LISTGDDCVS LLGGSQDILV SQVLCGPGHG
                                    *                        **
                                motif-I                  motif-II

             ....|....| ....|....| ....|....| ....|....| ....|....| ....|....| ....|....|
                   285        295        305        315        325        335        345
EnPoGaAn     LSIGSVGD-R SNNVVKNVTI EHSTVSNSEN AVRIKTI-SG ATGSVSEITY SNIVMSGISD YGVVIQQDYE
EnPoGaAp     LSIGSVGG-R SDNTVEDVTI SNSKVVNSQN GVRIKTV-YG ATGTVSNVKF EDITLSGITK YGLVVEQDYE
EnPoGaAo     LSIGSVGG-R SDNTVEDVTI SNSKVVNSQN GVRIKTV-YD ATGTVSNVKF EDITLSGITK YGLIVEQDYE
EnPoGaAa     LSIGSVGG-R SDNTVKNVTF VDSTIINSDN GVRIKTN-ID TTGSVSDVTY KDITLTSIAK YGIVVQQNYG
EnPoGAFm     LSIGSVGG-K SDNVVDGVQF LSSQVVNSQN GCRIKSN-SG ATGTINNVTY QNIALTNIST YGVDVQQDYL
UN027741     ISIGSLGKSG SWDQVYDVNV DGATISNTEN GVRIKTW-QG GHGFASNIVF ENVWMENVSN -PIIIDQYYC
UN029718     ISIGSLGKSG SWDQVYDVNV DGATISNTEN GVRIKTW-QG GHGFASNIVF ENVWMENVSN -PIIIDQYYC
UN040504     ISIGSLGKSG SWDQVYDVNV DGATISNTEN GVRIKTW-QG GHGFASNIVF ENVWMENVSN -PIIIDQYYC
UN045719     ISIGSLGKSG SWDQVYDVNV DGATISNTEN GVRIKTW-QG GHGFASNIVF ENVWMENVSN -PIIIDQYYC
UN048617     ISIGSLGKSG SWDQVYDVNV DGATISNTEN GVRIKTW-QG GHGFASNIVF ENVWMENVSN -PIIIDQYYC
UN062967     ISIGALGR-G GFDTVQNVSV QNSTLTGTMT GVRIKTW-QG GSGHARNIAY RRIKLVAVNN -PIIIDQFYC
UN068437     ISIGALGR-G GFDTVQNVSV QNSTLTGTMT GVRIKTW-QG GSGHARNIAY RRIKLVAVNN -PIIIDQFYC
UN070596     ISIGSLSKEM VEAGVQNVTV KTATFKGTQN GVRIKAWARA SNSYVRGVLF QHITMTSVQN -PIIIDQNYC
UN071326     ISIGSVGG-R SDNVVDGVTI ENSSISNSDN GVRIKTV-SG ATGSVNNVSY KGITLSNIAK YGIVIQQDYE
UN093239     ISIGSLGKEM EEAGVQNVTV KTATFKGTQN GVRIKAWARA SNSYVRGVLF QHITMTSVQN -PIIIDQNYC
UN097465     ISIGSVGG-R SDNVVDGVTI ENSSISNSDN GVRIKTV-SG ATGSVNNVSY KGITLSNIAK YGIVIQQDYE
UN101273     ISIGSLGNHN SVGIVTRVAV DTAVLRQTTN GVRIKTY-QG GSGQVSSVSY KNVRMEEVAN -PIIIDQFYC
UN153232     ISVGSLGKYE DEADVRRITV RHTTFVGTTN GVRIKTW--- ---------- ---------- ----------

        motif-III                                 motif-IV
```

Supplementary Figure S9. Multiple sequence alignment of polygalacturonase enzymes (members of GH28 family) from phytopathogenic fungi and the mistletoe *Psittacanthus schiedeanus*. The black line in the figure indicates the four conserved motifs (motif I: SPNTDG; II: GDDC; III: CGPGHGISIGSLG; and IV: RIK). The highly conserved amino acid residues which form the cleft of the active site are indicated by red-colored shading. The asterisk "*" highlights the catalytic residues (acid residues; Asp) which make possible the hydrolysis which occurs in the active site. Abbreviations [EnPoGaAp, EnPoGaAo, EnPoGaAn, EnPoGaAa and EnPoGaFm: endo-polygalacturonases enzymes from *Aspergillus parasiticus* (Uniprot: P49575), *A. oryzae* (Uniprot: P35335), *A. niger* (PDB ID: 1CZF), *A. aculeatus* (PDB ID: 1IA5), and *Fusarium moniliforme* (PDB ID: 1HG8), respectively].

```
            ....|....| ....|....| ....|....| ....|....| ....|....| ....|....| ....|....|
                5          15         25         35         45         55         65
EβD14MNs    ---------- ---------- -------KVP KGFVTTE--G DHFKLDGKDF YFAGSNAYYF PF-NDQPD--
EβD14MTp    ---------- ---------- MGSSHHHHHH SSGLVPR--G SHM-LNGKEF RFIGSNNYYM HY-KSNRM--
EβD14MPa    ---------- ---------- MGFLPQAQGG GAAASAKVSG TRFVIDGKTG YFAGTNSYWI GFLTNNRD--
EβD14MTr    ---------- ---------- ---------A SSFVTIS--G TQFNIDGKVG YFAGTNCYWC SFLTNHAD--
EβD14MAn    ---------- ---------- ---------- -SFASTS--G LQFTIDGETG YFAGTNSYWI GFLTDNAD--
UN035485    ---MKVLVLV ILMGLAVIHK DGFVTEADAW EGFIGTR--G VQFVLNGSPF YSNGFNAYWL MAVASDPTQR
UN039898    ---MKVLVLV ILMGLAVIHK DGFVTEADAW EGFIGTR--G VQFVLNGSPF YSNGFNAYWL MAVASDPTQR
UN051984    MLTTLGLCRV CAFICAIVAA AGDEAMLAES EGFVGTS--G SNFVVNRSPY MFNGFNSYWM MTVASDPSQR
UN066323    ---------V LLLPLLLIQQ QPSFGGAEAA GGFVTTK--G TQLMLNGSPF YANGFNAYWF MLIAADPSQR

            ....|....| ....|....| ....|....| ....|....| ....|....| ....|....| ....|....|
                75         85         95        105        115        125        135
EβD14MNs    --IEKGMTAA RAAGLTVFRT WGFNDKN-R- ----TYIPTG LPQYGNEGAG DPTNTVFQWF EADGTQTIDV
EβD14MTp    --IDSVLESA RDMGIKVLRI WGFLDGESYC RDKNTYMHPE PGVFGVPEGI SN-------- AQNGFERLDY
EβD14MPa    --VDTTLDHI ASSGLKILRV WGFNDVN-N- ----QPSGNT VWFQRLASSG SQINT----- GPNGLQRLDY
EβD14MTr    --VDSTFSHI SSSGLKVVRV WGFNDVN-T- ----QPSPGQ IWFQKLSATG STINT----- GADGLQTLDY
EβD14MAn    --VDLVMGHL KSSGLKILRV WGFNDVT-S- ----QPSSGT VWYQLHQDGK STINT----- GADGLQRLDY
UN035485    STVSAAFRQA TSHGLTVART WAFSDGG-Y- ----NPLQYS PGSY------ ---------- NEQMFKGLDF
UN039898    STVSAAFRQA TSHGLTVART WAFSDGG-Y- ----NPLQYS PGSY------ ---------- NEQMFKGLDF
UN051984    YKVSNVFRDA AAAGLSVCRT WAFSDGG-S- ----QALQSS PGVY------ ---------- GEKVFQGLDF
UN066323    QLVSSAYEAA SGHHLAIART WAFSDGGTY- ----NPLQTA PGIY------ ---------- NEVTFQGLDF

            ....|....| ....|....| ....|....| ....|....| ....|....| ....|....| ....|....|
               145        155        165        175        185        195        205
EβD14MNs    SPFDKVVDSA TKTGIKLIVA LTNNWADYGG MDVYTVNLGG -----KYHDD FYTVPKIKEA FKRYVKAMVT
EβD14MTp    -----TIAKA KELGIKLIIV LVNNWDDFGG MNQYVRWFGG -----THHDD FYRDERIKEE YKKYVSFLIN
EβD14MPa    -----LVRSA ETRGIKLIIA LVNYWDDFGG MKAYVNAFGG ------TKES WYTNARAQEQ YKRYIQAVVS
EβD14MTr    -----VVQSA EQHNLKLIIP FVNNWSDYGG INAYVNAFGG ------NATT WYTNTAAQTQ YRKYVQAVVS
EβD14MAn    -----VVSSA EQHDIKLIIN FVNYWTDYGG MSAYVSAYGG -----SGETD FYTSDTMQSA YQTYIKTVVE
UN035485    -----VVAEA GTYGIKLVLS LVNNYDNLGG KKQYVSWGRN QGQSLTSDDD FFTNQLVRGF YKDHVKTVLN
UN039898    -----VVAEA GTYGIKLVLS LVNNYDNLGG KKQYVSWGRN QGQSLTSDDD FFTNQLVRGF YKDHVKTVLN
UN051984    -----VIWEA KKHNVRLILS LVNNYKDYGG RSQYVSWARN LGVSVNHDDD FFTNAAVKGY YKNHVKTVLT
UN066323    -----VVAEA GKYGLKLILS LVNNYNDFGG KAQYVEWARN QGQIISSDDG FFTNSLVKGF YKNHIKAVLT

            ....|....| ....|....| ....|....| ....|....| ....|....| ....|....| ....|....|
               215        225        235        245        255        265        275
EβD14MNs    R--------Y RDSEAILAWE LANEARCGAD GTRNLPRSEK GCTTETVTGW IEEMSAYVKS LDGNHLVTWG
EβD14MTp    HVNVYTGVPY REEPTIMAWE LANELRCETD ---------- -KSGNTLVEW VKEMSSYIKS LDPNHLVAVG
EβD14MPa    R--------Y VNSPAIFAWE LANEPRCKG- ---------- -CNTNVIFNW ATQISDYIRS LDKDHLITLG
EβD14MTr    R--------Y ANSTAIFAWE LGNEPRCNG- ---------- -CSTDVIVQW ATSVSQYVKS LDSNHLVTLG
EβD14MAn    R--------Y SNSSAVFAWE LANEPRCPS- ---------- -CDTSVLYNW IEKTSKFIKG LDADRMVCIG
UN035485    RYNTMTGILY KDDPTIMAWE LMNEPRCTSD ---------- -PSGKTIQAW IMEMASHVKS IDSNHLLEVG
UN039898    RYNTMTGILY KDDPTIMAWE LMNEPRCTSD ---------- -PSGKTIQAW IMEMASHVKS IDSNHLLEVG
UN051984    RINTFTRIAY KDDPTIMAWE LMNEPRCQLD ---------- -VSGKTVNGW IQEMAPYVKS IDSKHLLEVG
UN066323    RVNTVTKVAY KDDPTIMAWE LMNEPRCPSD ---------- -PSGKTVQDW IAEMSSYLKS IDSKHLLEAG
                                     *

            ....|....| ....|....| ....|....| ....|....| ....|....| ....|....| ....|....|
               285        295        305        315        325        335        345
EβD14MNs    GEGGF----- -NRGEDEEDG F-YNGADGGD FDRELGLRNV DFGTMHLYPD WW-------S KSIEWSNQWI
EβD14MTp    DE-GFFSNYE GFKPYGGEAE WAYNGWSGVD WKKLLSIETV DFGTFHLYPS HW---GVSPE NYAQWGAKWI
EβD14MPa    DE-GF----- ---GLPGQTT YPYQYGEGTD FVKNLQIKNL DFGTFHMYPG HW-------G VPTSFGPGWI
EβD14MTr    DE-GL----- --GLSTGDGA YPYTYGEGTD FAKNVQIKSL DFGTFHLYPD SW-------G TNYTWGNGWI
EβD14MAn    DE-GF----- -GLNIDSDGS YPYQFSEGLN FTMNLDIDTI DFGTLHLYPD SW-------G TSDDWGNGWI
UN035485    LE-GFYGEST PQKMRLNPG- FNV----GTD FIANNQIAGI DFATAHSYPD QWLS-NTDDQ SQLSFLNNWL
UN039898    LE-GFYGEST PQKMRLNPG- FNV----GTD FIANNQIAGI DFATAHSYPD QWLS-NTDDQ SQLSFLNNWL
UN051984    LE-GFYGDSK PEREQSNPSG LLY----GTD FITNNQIKEI DFTTIHAYPE VWLP-NENGS VQRAFVQRWM
UN066323    LE-GFYGQ-- -AQQQNNPNT WPV----GTD FLADNQIPSI DFATVHSYPD QWLPNNTSNE AQLQFLNKWI

            ....|....| ....|....| ....|....| ....|....| ....|....| ....|....| ....|....|
               355        365        375        385        395        405        415
EβD14MNs    HDHAASGRAA NK-PVVLEEY GWMT-DKGRL DQLGQVKNET RLEVVGGWQK IA---IQEKL A--GDMYWQF
EβD14MTp    EDHIKIAKEI GK-PVVLEEY GIPKSAP--- --------VN RTAIYRLWND LV---YDLGG D--GAMFWML
EβD14MPa    KDHAAACRAA GK-PCLLEEY GYESDRC--- ---------- --NVQKGWQQ ASRELSRDGM S--GDLFWQW
EβD14MTr    QTHAAACLAA GK-PCVFEEY GAQQNPC--- ---------- --TNEAPWQT TS--LTTRGM G--GDMFWQW
EβD14MAn    TAHGAACKAA GK-PCLLEEY GVTSNHC--- ---------- --SVEGAWQK TA--LSTTGV G--ADLFWQY
UN035485    SSHIHDTESI LRKPLLLAEF GKSTKDPGFS D---SQRESM FNSVYFNVYS SA----RTGG AAAGGLFWQL
UN039898    SSHIHDTESI LRKPLLLAEF GKSTKDPGFS D---SQRESM FNSVYFNVYS SA----RTGG AAAGGLFWQL
UN051984    AVHAADAAAA KK-PLVFGEF GRSKKVAGYS L---QARESY LNEIFSTINK MAQQKSRTRG SIVGSLVWQI
UN066323    KVHIQDAQQT LKKPVLFAEF GKSSNQPGYT V---AERNEL YTTVYSGIYA SA----ESGG AAVGGLFWQL
                               *
```

Supplementary Figure S10. Multiple sequence alignment of mannanases enzymes (members of GH5-7 family) from phytopathogenic fungi and the mistletoe *Psittacanthus schiedeanus*. The highly conserved and functional amino acid residues which form the slot-like pocket are indicated by red-colored shading. The asterisk "*" highlights the catalytic residues (acid residues; Glu) which make possible the hydrolysis which occurs in the active site. Abbreviations [EβD14MNs, EβD14MTp, EβD14MPa, EβD14MTr, EβD14MAn: endo-1,4-beta-D-mannanase from *Neurospora sitophila* (PDB ID: 4AWE), *Thermotoga petrophila* RKU-1 (PDB ID: 3PZ9), *Podospora anserina* (PDB ID: 3ZIZ), *Trichoderma reesei* (PDB ID: 1QNO), and *Aspergillus niger* BK01 (PDB ID: 3WH9), respectively].
